# Predicting the impact of patient and private provider behavior on diagnostic delay for pulmonary tuberculosis patients in India: A simulation modeling study

**DOI:** 10.1371/journal.pmed.1003039

**Published:** 2020-05-14

**Authors:** Sarang Deo, Simrita Singh, Neha Jha, Nimalan Arinaminpathy, Puneet Dewan

**Affiliations:** 1 Indian School of Business, Hyderabad, India; 2 Kellogg School of Management, Northwestern University, Evanston, Illinois, United States of America; 3 Kenan-Flagler Business School, University of North Carolina, Chapel Hill, North Carolina, United States of America; 4 School of Public Health, Imperial College London, London, United Kingdom; 5 Bill & Melinda Gates Foundation, Seattle, Washington, United States of America; UniversitatsKlinikum Heidelberg, GERMANY

## Abstract

**Background:**

Tuberculosis (TB) incidence in India continues to be high due, in large part, to long delays experienced by patients before successful diagnosis and treatment initiation, especially in the private sector. This diagnostic delay is driven by patients’ inclination to switch between different types of providers and providers’ inclination to delay ordering of accurate diagnostic tests relevant to TB. Our objective is to quantify the impact of changes in these behavioral characteristics of providers and patients on diagnostic delay experienced by pulmonary TB patients.

**Methods and findings:**

We developed a discrete event simulation model of patients’ diagnostic pathways that captures key behavioral characteristics of providers (time to order a test) and patients (time to switch to another provider). We used an expectation-maximization algorithm to estimate the parameters underlying these behavioral characteristics, with quantitative data encoded from detailed interviews of 76 and 64 pulmonary TB patients in the 2 Indian cities of Mumbai and Patna, respectively, which were conducted between April and August 2014. We employed the estimated model to simulate different counterfactual scenarios of diagnostic pathways under altered behavioral characteristics of providers and patients to predict their potential impact on the diagnostic delay. Private healthcare providers including chemists were the first point of contact for the majority of TB patients in Mumbai (70%) and Patna (94%). In Mumbai, 45% of TB patients first approached less-than-fully-qualified providers (LTFQs), who take 28.71 days on average for diagnosis. About 61% of these patients switched to other providers without a diagnosis. Our model estimates that immediate testing for TB by LTFQs at the first visit (at the current level of diagnostic accuracy) could reduce the average diagnostic delay from 35.53 days (95% CI: 34.60, 36.46) to 18.72 days (95% CI: 18.01, 19.43). In Patna, 61% of TB patients first approached fully qualified providers (FQs), who take 9.74 days on average for diagnosis. Similarly, immediate testing by FQs at the first visit (at the current level of diagnostic accuracy) could reduce the average diagnostic delay from 23.39 days (95% CI: 22.77, 24.02) to 11.16 days (95% CI: 10.52, 11.81). Improving the diagnostic accuracy of providers per se, without reducing the time to testing, was not predicted to lead to any reduction in diagnostic delay. Our study was limited because of its restricted geographic scope, small sample size, and possible recall bias, which are typically associated with studies of patient pathways using patient interviews.

**Conclusions:**

In this study, we found that encouraging private providers to order definitive TB diagnostic tests earlier during patient consultation may have substantial impact on reducing diagnostic delay in these urban Indian settings. These results should be combined with disease transmission models to predict the impact of changes in provider behavior on TB incidence.

## Abstract 2

Sarang Deo and co-workers study providers' approaches to tuberculosis diagnosis in India.

## Author summary

**Why was this study done?:**

- India contributes more than a quarter of the 10 million global tuberculosis (TB) cases every year.
- Several studies capture long, circuitous care pathways followed by TB patients until their diagnosis. However, these studies do not quantify the link between diagnostic delay and underlying patient and provider behavior characteristics.

**What did the researchers do and find?:**

- We developed a quantitative simulation model to estimate the impact of behavioral characteristics of patients and providers on diagnostic delay and estimated the parameters of this model using data from detailed interviews of 76 patients from Mumbai and 64 patients from Patna.
- We found that earlier test ordering by providers would yield a much larger reduction in diagnostic delay than increasing their diagnostic accuracy.

**What do these findings mean?:**

- Policy-makers and implementing agencies should encourage early test ordering behavior by providers to reduce diagnostic delay, and, consequently, to reduce disease transmission.

## Introduction

The success of the global fight against TB hinges critically on progress made in India, which accounted for more than a quarter of the estimated 10 million global tuberculosis (TB) cases in 2017 [1]. In response to this challenge, the Revised National Tuberculosis Control Programme reported substantial scale-up and intensification of its efforts over the period 2012–2017 [2]. Yet, the estimated incidence over this period decreased at an annual rate of only 1.5%, from around 220 cases per 100,000 in 2012 to 204 cases per 100,000 in 2017 [1,3]. In this environment, achieving the ambitious goal of the WHO End TB Strategy [4] requires large-scale engagement of private healthcare providers [5,6]: These providers treat more than half of the estimated TB cases [7,8] and are often the first point of contact even for patients treated in the public sector [9,10], but follow suboptimal diagnostic and treatment practices [11].

India’s private healthcare sector comprises a heterogenous and fragmented base of providers including those trained in the Western system of medicine (allopathy), those trained in indigenous systems of medicine (Ayurveda, Unani, Siddha, and homeopathy), and those with no formal training in medicine (informal or less-than-fully-qualified providers [LTFQs]) [12,13]. These private providers rarely order TB-specific microbiological tests such as smear microscopy and culture, especially in the first visit [14,15]. Instead, they often rely on empiric treatment: initiating patients on broad spectrum antibiotics and fluoroquinolones and following up with chest X-ray and blood tests if the patient’s symptoms do not improve [16,17]. Patients who are not diagnosed successfully or do not experience improvement in their condition frequently switch providers (a phenomenon informally described as “provider shopping”), and the cycle is likely to continue until the patient is eventually diagnosed and initiated on effective treatment [9,10,18–22]. Interactions between negative provider behaviors (long delay in test ordering, low diagnostic accuracy) and patient behavior (frequent switching) result in complex pathways and long diagnostic delays [10] and ultimately lead to high rates of TB incidence, as infectious TB patients mix with susceptible individuals in the community [23]. Designing provider engagement strategies that reduce TB incidence in a cost-effective manner will require identifying and prioritizing elements of provider behavior that have the largest impact on diagnostic delay.

Toward this end, we developed a comprehensive quantitative model of provider and patient behaviors in the pulmonary TB diagnostic pathway; here we quantify the impact of changing key aspects of these behaviors on diagnostic delay, and disentangle this impact from the impact of new, more accurate diagnostic tools.

## Methods

### Data Sources

We utilized data from a population-based 2-stage retrospective study that identified and interviewed self-reported pulmonary TB patients in 2 Indian cities, Mumbai and Patna [20,21]. These data were not originally collected for the purpose of the modeling analysis described here. The analysis plan was developed after all the instruments and interview guides had been finalized and while the data collection process was ongoing. The original studies involving data collection and patient interviews were approved by the ethics committee at the Foundation for Medical Research (IEC no. FMR/IEC/TB/01/2013).

Household surveys were conducted in 15 wards of the Municipal Corporation of Greater Mumbai with high burden of TB and high slum population, and 140 wards under the Patna Municipal Corporation and Danapur and Phulwari Sharif Municipal Councils. The survey of 14,250 participating households in Mumbai and 12,957 households in Patna led to the identification of 153 and 106 self-reported pulmonary TB patients, respectively, who either were being treated for TB or had finished their treatment in the past 6 months. These patients were followed up by trained public health researchers within 3–4 days. However, 77 patients in Mumbai and 45 patients in Patna could not be tracked even after 3 visits and hence were not interviewed. Each of the remaining 76 patients in Mumbai and 64 patients in Patna was interviewed by 2 researchers for 60–90 minutes. Interviews were conducted using a pretested open-ended interview schedule. The schedule was first developed in English, translated into local languages (Hindi and Marathi), and then back translated into English to check for consistency. The responses were coded according to a predesigned quantitative data sheet to construct patient pathways.

Each encoded pathway contained information on multiple stages of consultation for that patient, with each stage comprising multiple visits made to a single healthcare provider. For each stage of consultation, the dates of the first and the last visit and the tests ordered (if any) by the provider consulted during that stage were recorded. If the patient received a diagnosis in a particular stage of consultation, then the date of diagnosis and the specific diagnosis given (e.g., TB, multidrug-resistant TB, typhoid) were recorded. In addition, at every stage of consultation, the qualification of the provider (e.g., bachelor of medicine and bachelor of surgery [MBBS], doctor of medicine [MD], bachelor of Ayurvedic medicine and surgery [BAMS], informal provider) and the associated facility (e.g., municipal/private hospital, pharmacy) were recorded. Private providers with qualifications equivalent to MBBS or MD were classified as fully qualified providers (FQs), those with other qualifications or no qualifications were classified as LTFQs, and those operating a retail pharmacy were labeled as chemists. Providers associated with government/municipal facilities were classified as public providers. The original studies that reported the survey data follow a slightly different classification; they combine all providers other than public providers into a single category of private providers [20,21].

Three levels of data checks were undertaken for quality assurance. First, an inter-researcher exchange of interview audio files, notes, and quantitative data was conducted. Second, interviews undertaken by each set of 2 researchers were rechecked by 2 senior researchers of the team. Third, a final review of the interviews was conducted by an external consultant. More details can be found in the original studies [20,21].

### Model structure

**Fig 1:**
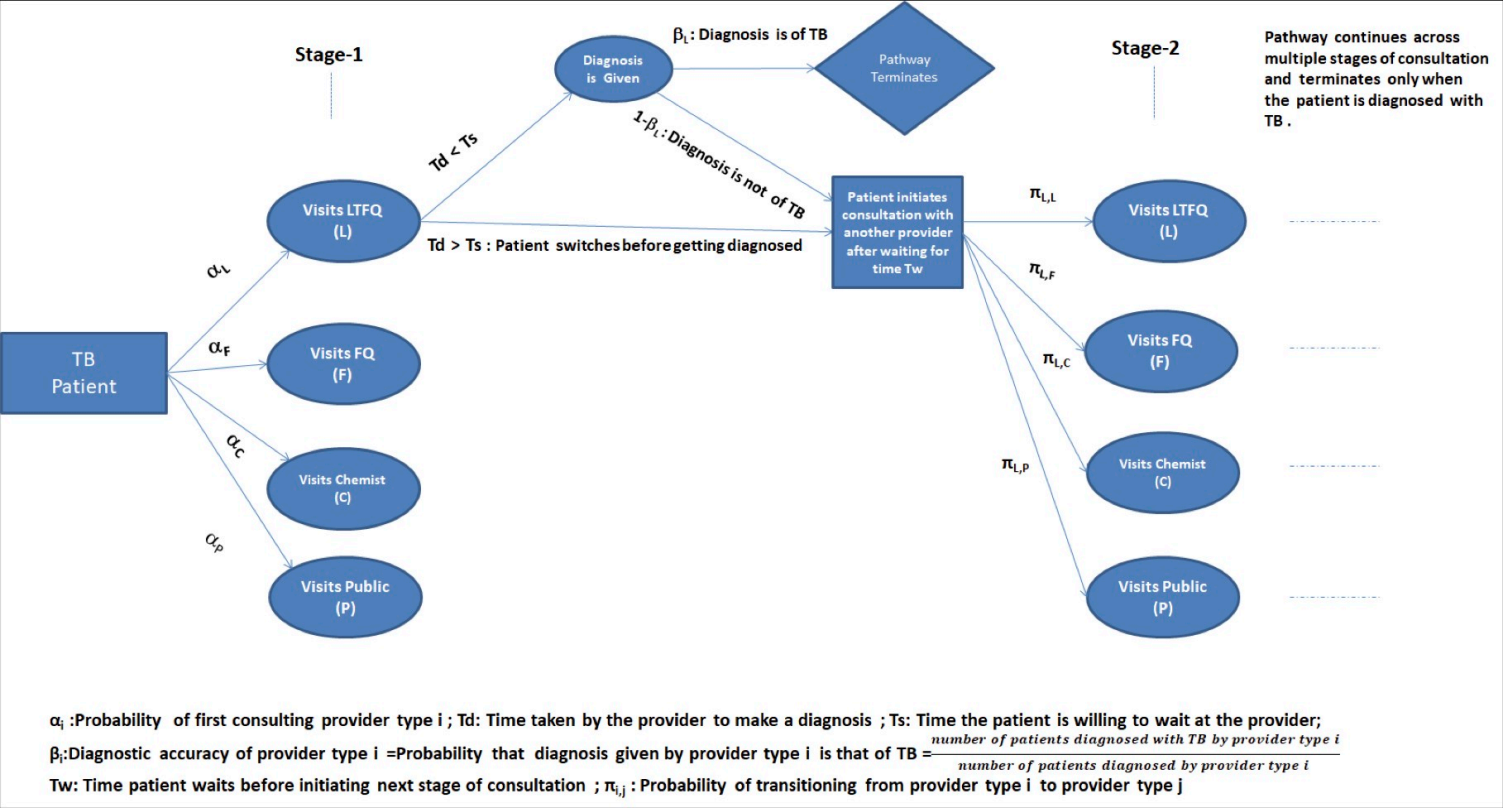
Schematic representation of the simulation model. FQ, fully qualified provider; LTFQ, less-than-fully-qualified provider; TB, tuberculosis.

We constructed a discrete event simulation model of TB patient pathways as a series of consultation stages consisting of 2 building blocks: (i) behavior of patients and providers within a single stage of consultation and (ii) transition of patients from one stage of consultation to the next (Fig 1).

In the model, at the beginning of the pathway, each patient seeking care for his/her symptoms chooses a provider of a particular category (LTFQ, FQ, chemist, public provider) for the first consultation with a certain probability (α). The patient is willing to continue in this consultation stage for a random duration, called time to switching (*T*_s_), which is exponentially distributed, with mean τ_s_. Similarly, the physician is willing to order a diagnostic test and/or give a clinical diagnosis after a random period of time, called time to diagnosis (*T*_d_), which is also exponentially distributed, with mean τ_d_. If *T*_d_ ≤ *T*_s_, then the patient receives diagnosis before switching to another provider, and if *T*_d_ > *T*_s_, the patient switches to another provider before receiving a diagnosis. With certain probability β, the patient is successfully diagnosed of TB, upon which the patient pathway terminates. If *T*_d_ > *T*_s_ or if the patient receives a diagnosis other than TB, i.e., fails to receive TB diagnosis (with probability 1 − β), then he/she waits for some time (*T*_w_) and initiates the next stage of consultation with a different provider. The probability (π) that the patient chooses a provider of a particular type for the next stage of consultation depends on the category of the provider consulted during the previous stage of consultation. In line with previous studies, assuming the accuracy of successful TB diagnosis by provider type allowed us to interpret previous diagnosis other than TB to be inaccurate and thereby β to be the diagnostic accuracy of the provider type with respect to TB. To replicate the maximum number of consultation stages in the data, i.e., the maximum number of providers visited by patients in each city (5 for Mumbai and 4 for Patna), we generated a final diagnosis in those stages for the few patients whose pathway did not terminate naturally by then (<2%).

### Analysis

We used the above model structure to derive the likelihood of observing the data as a function of the underlying parameters. We obtained the estimates of the model parameters—probability of first consultation (α), rate of diagnosis (τ_s/_τ_d_), rate of switching (1/τ_s_), diagnostic accuracy (β), and transition probabilities (π)—and the associated variance–covariance matrix using an expectation-maximization (EM) algorithm to account for missing data on provider qualifications [14,15]. We used these estimates to calculate the mean time to diagnosis (τ_d_), the mean time to switching (τ_s_), and the probability of receiving a diagnosis in a consultation stage for each category of provider (τ_s_/[τ_s_ + τ_d_]). We also calculated the probability of receiving a correct diagnosis as the product of the probability of receiving a diagnosis and the diagnostic accuracy of the provider at that stage, β(τ_s_/[τ_s_ + τ_d_]). See S1 Text for the expression of the likelihood function and details of the EM algorithm. Additionally, in response to reviewer suggestion, we estimated the model separately for a subsample of new patients and also estimated a model variant where the rate of switching and rate of diagnosis were allowed to vary across stages of consultation.

We conducted an in-sample validation of our model as follows. We simulated 10,000 patient pathways by randomly drawing parameter values from a multivariate normal distribution with the mean and variance–covariance matrix estimated above. We calculated the diagnostic delay and the number of stages of consultation for each simulated pathway and compared their mean and variance with those in the raw dataset using standard statistical tests. We also conducted a limited out-of-sample validation exercise due to the small sample size (S2 Text).

We varied the parameters of the validated base model to investigate the impact of early and accurate diagnosis on the diagnostic delay by simulating 4 sets of counterfactual scenarios: (i) time to diagnosis (i.e., delay in test ordering) of LTFQs reduced to 0, (ii) time to diagnosis of FQs reduced to 0, (iii) diagnostic accuracy of LTFQs increased to 100%, and (iv) diagnostic accuracy of FQs increased to 100%. Each set consisted of 5 individual scenarios, where we varied the coverage of providers, i.e., the percentage of providers who exhibit the improved behavior, from 20% to 100% in increments of 20%. For each of these scenarios, we simulated 10,000 patient pathways and compared the mean and variance of the diagnostic delay and the number of stages of consultation until correct diagnosis with those in the base model. We used R version 3.3.1 (2016-06-21) for modeling, estimation, and counterfactual simulation, and R package ‘stats’ for the in-sample statistical validation of our results. Programming code for estimation and counterfactual simulation is available from the authors on request.

This study is reported as per the STRESS-DES guidelines (S1 Checklist). Datasets used for the study are available in Dryad (https://doi.org/10.5061/dryad.bt36t [Mumbai] and https://doi.org/10.5061/dryad.2ngf1vhjn [Patna]).

## Results

### Data description

Demographic characteristics of the study participants are presented in earlier studies [20,21] and are summarized in Table 1.

**Table 1:** Demographics and characteristics of the study sample.

Characteristic	Mumbai *n* (%)	Patna *n* (%)
**Age (years)**		
≤17	8 (10.5%)	19 (30%)
≥18	68 (89.5%)	45 (70%)
**Sex**		
Male	46 (61%)	33 (52%)
Female	30 (39%)	31 (48%)
**Occupation**		
Unemployed	31 (41%)	6 (9%)
Salaried	11 (14%)	9 (14%)
Self-employed	10 (13%)	10 (16%)
Daily wage/casual labor	6 (8%)	6 (9%)
Housewife	9 (12%)	17 (27%)
Student	9 (12%)	13 (20%)
Not available	—	3 (5%)
**Education**		
Illiterate	14 (18%)	22 (34%)
Primary	8 (11%)	7 (11%)
Secondary	32 (42%)	7 (11%)
Senior secondary	16 (21%)	8 (13%)
Graduate or above	4 (5%)	3 (5%)
Not available	2 (3%)	2 (3%)
**Monthly household income (INR)**		
<10,000	26 (35%)	44 (69%)
10,000 to 30,000	24 (31%)	15 (23%)
≥30,000	4 (5%)	3 (5%)
Not available	22 (29%)	2 (3%)
**History of tuberculosis**		
New case	43 (57%)	49 (77%)
Retreatment	33 (43%)	15 (23%)

The sample from Mumbai comprised of 76 patients, whereas the sample from Patna consisted of 64 patients.

INR, Indian rupees.

Table 2 displays the following entities related to the patient pathway, aggregated across all stages of consultation for each provider type: (i) fraction of patients receiving a diagnosis; (ii) of those, fraction of patients receiving correct diagnosis; and (iii) average time spent by patients until TB diagnosis. Finally, it also displays the fraction of patients that chose a provider of that type as the first point of contact. For Mumbai, average time spent by patients differed across provider types (1-way ANOVA; *p* = 0.06). Similarly, the fraction of patients receiving a diagnosis as well as the accuracy of diagnosis were different across provider types (chi-squared test; *p* < 0.001 and *p* = 0.03, respectively). For Patna, average time spent by patients likewise differed across provider types (1-way ANOVA; *p* = 0.02). Similarly, the fraction of patients receiving a diagnosis was different across provider types (chi-squared test; *p* < 0.001), but not the fraction of patients receiving a correct diagnosis (chi-squared test; *p* = 1). Finally, the diagnostic accuracy of chemists and LTFQs could not be estimated in Patna, and that of LTFQs could not be estimated in Mumbai, as there were no diagnoses by those types of providers in those respective cities.

**Table 2:** Aggregated descriptive statistics of patient pathway survey data.

Provider type	Patients receiving diagnosis, *n* (%)		Patients receiving correct diagnosis, *n* (%)		Average time patients spent until diagnosis (days), mean (95% CI)		First point of contact, *n* (%)	
	Mumbai	Patna	Mumbai	Patna	Mumbai	Patna	Mumbai	Patna
Public	36 (68%)	0.96	34 (94%)	1.00	8.62 (4.91, 12.34)	5.43 (3.28, 7.59)	23 (30%)	4 (6%)
FQ	22 (71%)	0.66	20 (91%)	0.97	15.52 (5.87, 21.56)	10.47 (5.14, 15.81)	7 (9%)	33 (52%)
LTFQ	16 (43%)	0.00	11 (69%)	NA	21.86 (10.47, 32.26)	15.00 (1.95, 28.05)	25 (33%)	4 (6%)
Chemist	0 (0%)	0.00	NA	NA	25.00 (8.17, 41.83)	22.17 (12.20, 32.03)	9 (12%)	16 (25%)
Unknown	11 (38%)	0.45	11 (100%)	0.80	25.69 (7.85, 43.53)	27.27 (2.70, 51.84)	12 (16%)	7 (11%)

FQ, fully qualified provider; LTFQ, less-than-fully-qualified provider; NA, not applicable.

Table 3 and Table 4 provide further breakdown of the first 3 entities in Table 2, along with the number of patients visiting each type of provider by stage of consultation, for Mumbai and Patna, respectively. For both cities, we found that the fraction of patients receiving a diagnosis increased in later stages of consultation, whereas the fraction of patients receiving a correct diagnosis remained relatively steady and high overall across public providers, FQs, and LTFQs. For Mumbai, the point estimate of the average time spent by patients with providers was generally higher in the first stage than in the subsequent stages.

**Table 3:** Stage-wise descriptive statistics of patient pathway data: Mumbai.

Provider type	Number of patients, *n* (%)					Patients receiving diagnosis, *n* (%)					Patients receiving correct diagnosis, *n* (%)					Average time patients spent until diagnosis (days), mean (95% CI)				
	1	2	3	4	5	1	2	3	4	5	1	2	3	4	5	1	2	3	4	5
Public	23 (30%)	17 (22%)	7 (9%)	3 (4%)	3 (4%)	13 (57%)	14 (82%)	3 (43%)	3 (100%)	3 (100%)	13 (100%)	13 (93%)	3 (100%)	2 (67%)	3 (100%)	13.6 (5.5, 21.6)	5.6 (3.8, 7.4)	3.1 (2.2, 4.0)	5 (0.1, 9.9)	4.3 (0.0, 9.91)
FQ	7 (9%)	15 (20%)	5 (7%)	4 (5%)	0 (0%)	3 (43%)	11 (73%)	4 (80%)	4 (100%)	NA	2 (67%)	11 (100%)	3 (75%)	4 (100%)	NA	20.6 (0.0, 47.0)	12.9 (6.0, 19.7)	27.8 (0.0, 72.6)	1.2 (0.0, 2.5)	NA
LTFQ	25 (33%)	7 (9%)	2 (3%)	3 (4%)	0 (0%)	8 (32%)	5 (71%)	2 (100%)	1 (33%)	NA	5 (63%)	4 (80%)	1 (50%)	1 (100%)	NA	29.2 (13.1, 45.3)	8.1 (4.9, 11.4)	4.0 (4.0, 4.0)	4.7 (0.0, 9.9)	NA
Chemist	9 (12%)	0 (0%)	0 (0%)	0 (0%)	0 (0%)	0 (0%)	NA	NA	NA	NA	NA	NA	NA	NA	NA	25.0 (8.2, 41.8)	NA	NA	NA	NA
Unknown	12 (16%)	12 (16%)	5 (7%)	0 (0%)	0 (0%)	5 (42%)	4 (33%)	2 (40%)	NA	NA	5 (100%)	4 (100%)	2 (100%)	NA	NA	22.9 (0.0, 46.9)	25.5 (0.0, 58.1)	32.8 (0.0, 78.2)	NA	NA

FQ, fully qualified provider; LTFQ, less-than-fully-qualified provider; NA, not applicable.

**Table 4:** Stage-wise descriptive statistics of patient pathway data: Patna.

	Number of patients, *n* (%)					Patients receiving diagnosis, *n* (%)					Patients receiving correct diagnosis, *n* (%)					Average time patients spent until diagnosis (days), mean (95% CI)				
	1	2	3	4	5	1	2	3	4	5	1	2	3	4	5	1	2	3	4	5
Public	4 (6%)	15 (23%)	3 (5%)	1 (2%)	0 (0%)	4 (100%)	14 (93%)	3 (100%)	1 (100%)	NA	4 (100%)	14 (100%)	3 (100%)	1 (100%)	NA	3.5 (0.5, 6.6)	4.6 (2.4, 6.8)	7.3 (0.7, 14.0)	NA	NA
FQ	33 (52%)	15 (23%)	8 (13%)	3 (5%)	0 (0%)	23 (70%)	6 (40%)	7 (88%)	3 (100%)	NA	23 (100%)	6 (100%)	6 (86%)	3 (100%)	NA	9.1 (2.5, 15.7)	19.4 (5.0, 33.8)	2.4 (0.9, 3.9)	2.3 (1.7, 3.0)	NA
LTFQ	4 (6%)	0 (0%)	0 (0%)	0 (0%)	0 (0%)	0 (0%)	NA	NA	NA	NA	NA	NA	NA	NA	NA	15.0 (2.0, 28.1)	NA	NA	NA	NA
Chemist	16 (25%)	1 (2%)	1 (2%)	1 (2%)	0 (0%)	0 (0%)	NA	NA	NA	NA	NA	NA	NA	NA	NA	23.1 (11.9, 34.3)	NA	NA	NA	NA
Unknown	7 (11%)	3 (5%)	1 (2%)	0 (0%)	0 (0%)	3 (43%)	2 (67%)	0 (0%)	NA	NA	3 (100%)	1 (50%)	NA	NA	NA	32.6 (0.0, 71.1)	17.7 (0.0, 36.5)	NA	NA	NA

FQ, fully qualified provider; LTFQ, less-than-fully-qualified provider; NA, not applicable.

### Parameter estimation

The patient pathways of TB patients diagnosed in Mumbai and Patna show different patterns of first consultation and switching between providers (Table 5). In Mumbai, the probability of LTFQs and public providers being the first point of contact for patients was 0.45 (95% CI: 0.33, 0.57) and 0.30 (95% CI: 0.15, 0.47), respectively. In Patna, the probability of FQs and LTFQs being the first point of contact was 0.61 (95% CI: 0.45, 0.77) and 0.25 (95% CI: 0.00, 0.50), respectively. A patient in Mumbai visiting an LTFQ as the first point of contact switched to a public provider with probability 0.46 (95% CI: 0.22, 0.70) and to an FQ with probability 0.39 (95% CI: 0.20, 0.60). In comparison, a patient in Patna first visiting an FQ switched to another FQ with probability 0.76 (95% CI: 0.58, 0.94) and to a public provider with probability 0.24 (95% CI: 0.06, 0.42).

**Table 5:** Probabilities of first consultation and probability of switching across provider types.

First provider type	City	Switching provider type (π), mean (95% CI)				First consultation (α), mean (95% CI)
		Public	FQ	LTFQ	Chemist	
**Public**	Mumbai	0.47 (0.24, 0.75)	0.32 (0.14, 0.53)	0.21 (0.06, 0.39)	NA	0.30 (0.15, 0.47)
	Patna	1.00 (1.00, 1.00)	NA	NA	NA	0.08 (0.00, 0.30)
**FQ**	Mumbai	0.45 (0.16, 0.81)	0.43 (0.19, 0.73)	0.11 (0.00, 0.31)	NA	0.13 (0.06, 0.23)
	Patna	0.24 (0.06, 0.42)	0.76 (0.58, 0.94)	NA	NA	0.61 (0.45, 0.77)
**LTFQ**	Mumbai	0.46 (0.22, 0.70)	0.39 (0.20, 0.60)	0.15 (0.03, 0.31)	NA	0.45 (0.33, 0.57)
	Patna	0.50 (0.00, 1.00)	0.30 (0.00, 0.92)	0.20 (0.00, 0.70)	NA	0.25 (0.00, 0.50)
**Chemist**	Mumbai	0.11 (0.00, 0.58)	0.44 (0.17, 0.77)	0.44 (0.17, 0.77)	NA	0.12 (0.06, 0.19)
	Patna	0.44 (0.16, 0.72)	0.44 (0.20, 0.68)	0.00 (0.00, 0.00)	0.11 (0.00, 0.26)	0.06 (0.15, 0.35)

FQ, fully qualified provider; LTFQ, less-than-fully-qualified provider; NA, not applicable.

The estimated average time to diagnosis and switching are shown in Table 6. The estimates of the underlying rates of diagnosis and switching, along with their respective confidence intervals, are available in S1 Table. In Mumbai, an LTFQ takes 28.71 days, and a public provider takes 11.44 days, on average to provide a diagnosis. Similarly, in Patna, an FQ takes 9.74 days on average to provide a diagnosis. Average time to diagnosis could not be estimated for chemists in Patna because none of the patients in the sample received a diagnosis from a chemist. In Mumbai, mean time to switching was 18.59 days (95% CI: 10.77, 27.99) from an LTFQ and 22.96 days (95% CI: 15.92, 31.50) from an FQ. Similarly, in Patna, mean time to switching was estimated to be 18.24 days (95% CI: 10.59, 25.88) from an FQ and 8.30 days (95% CI: 5.78, 10.82) from a chemist. Based on these values, the calculated probability of receiving a diagnosis (correct or incorrect) at an LTFQ in Mumbai was 0.38 (95% CI: 0.24, 0.54) and at an FQ in Patna was 0.65 (95% CI: 0.53, 0.76). The corresponding probabilities for other types of providers were 0.63 (95% CI: 0.61, 0.75) for FQs and 0.68 (95% CI: 0.61, 0.75) for public providers in Mumbai and 0.96 (95% CI: 0.94, 0.97) for public providers in Patna. For the model variant that allowed rates of diagnosis and switching to be stage-dependent, both showed an increasing pattern across consultation stages in Mumbai (S2 Table).

**Table 6:** Time to diagnosis and switching (days) and probability of receiving diagnosis.

Provider type	Time to diagnosis in days (τ_d_), mean (95% CI)		Time to switching in days (τ_s_), mean (95% CI)		Probability of receiving a diagnosis (τ_s_/[τ_s_ + τ_d_]), mean (95% CI)	
	Mumbai	Patna	Mumbai	Patna	Mumbai	Patna
Public	11.44 (9.05, 14.13)	5.57 (4.12, 7.03)	24.23 (18.75, 30.64)	123.52 (85.98, 161.06)	0.68 (0.61, 0.75)	0.96 (0.94, 0.97)
FQ	13.53 (9.43, 18.32)	10.07 (6.45, 13.68)	22.96 (15.92, 31.50)	18.24 (10.59, 25.88)	0.63 (0.52, 0.73)	0.65 (0.53, 0.76)
LTFQ	38.71 (0.28, 61.60)	NA	18.59 (10.77, 27.99)	21.78 (14.21, 29.34)	0.38 (0.24, 0.54)	NA
Chemist	NA	NA	16.33 (12.01, 21.33)	8.3x (5.78, 10.82)	NA	NA

FQ, fully qualified provider; LTFQ, less-than-fully-qualified provider; NA, not applicable.

Table 7 displays the diagnostic accuracy of providers and the probability of receiving a correct diagnosis. The lowest accuracy was that of LTFQs in Mumbai, about 75%. All other providers in both cities had a very high accuracy of around 95%. The probability of receiving a correct diagnosis was only 29% for LTFQs in Mumbai and 59%–64% for FQs in Mumbai and Patna as well as public providers in Mumbai. Remarkably, the probability of receiving a correct diagnosis was more than 95% for public providers in Patna. None of the estimated parameters were statistically different for new patients (S3–S5 Tables).

**Table 7:** Diagnostic accuracy and probability of receiving a correct diagnosis.

Provider type	Diagnostic accuracy (β), mean (95% CI)		Probability of receiving a correct diagnosis (β × [τ_s_/(τ_s_ + τ_d_)]), mean (95% CI)	
	Mumbai	Patna	Mumbai	Patna
Public	0.94 (0.90, 0.98)	1.00 (1.00, 1.00)	0.64 (0.57, 0.71)	0.96 (0.94, 0.97)
FQ	0.93 (0.87, 0.99)	0.95 (0.89, 1.00)	0.59 (0.48, 0.69)	0.61 (0.49, 0.73)
LTFQ	0.75 (0.55, 0.95)	1.00 (1.00, 1.00)	0.29 (0.15, 0.44)	NA
Chemist	NA	NA	NA	NA

FQ, fully qualified provider; LTFQ, less-than-fully-qualified provider; NA, not applicable.

### Model validation

Table 8 provides a comparison of raw data and simulated pathways on 2 outcomes, diagnostic delay and number of providers consulted before the first correct diagnosis. For each of the metrics, both mean and variance are not statistically different between the raw data and simulated pathways for both Mumbai and Patna, thereby demonstrating the internal validity of our simulation model. Results from out-of-sample validation also did not reveal any significant difference between model predictions and actual data (S7 Text).

**Table 8:** Model validation: Comparison of original data and simulated pathways.

Outcome	Mumbai			Patna		
	Original data, mean (95% CI)	Simulated data, mean (95% CI)	*p*-Value	Original data, mean (95% CI)	Simulated data, mean (95% CI)	*p*-Value
**Diagnostic delay**						
Mean	35.75 (25.34, 46.16)	35.52 (34.60, 36.46)	0.97	23.11 (14.83, 31.39)	23.39 (22.77, 24.01)	0.99
Variance	2,142.3 (1,593.3, 3,034.8)	2,260.1 (2,198.7, 2,324.1)	0.75	1,142.6 (829.1, 1,676.0)	1,012.8 (985.3, 1,041.4)	0.49
**Number of providers consulted**						
Mean	2.09 (1.85, 2.34)	2.05 (2.04, 2.06)	0.76	1.79 (1.57, 2.02)	1.77 (1.75, 1.79)	0.82
Variance	1.18 (0.88, 1.67)	1.31 (1.27, 1.34)	0.54	0.84 (0.61, 1.23)	0.74 (0.72, 0.76)	0.48

*p-*Value for comparison of mean is based on 2-sample Welch test. *p-*Value for comparison of variance is based on Bartlett *K*-squared test.

### Counterfactual simulation

**Fig 2:**
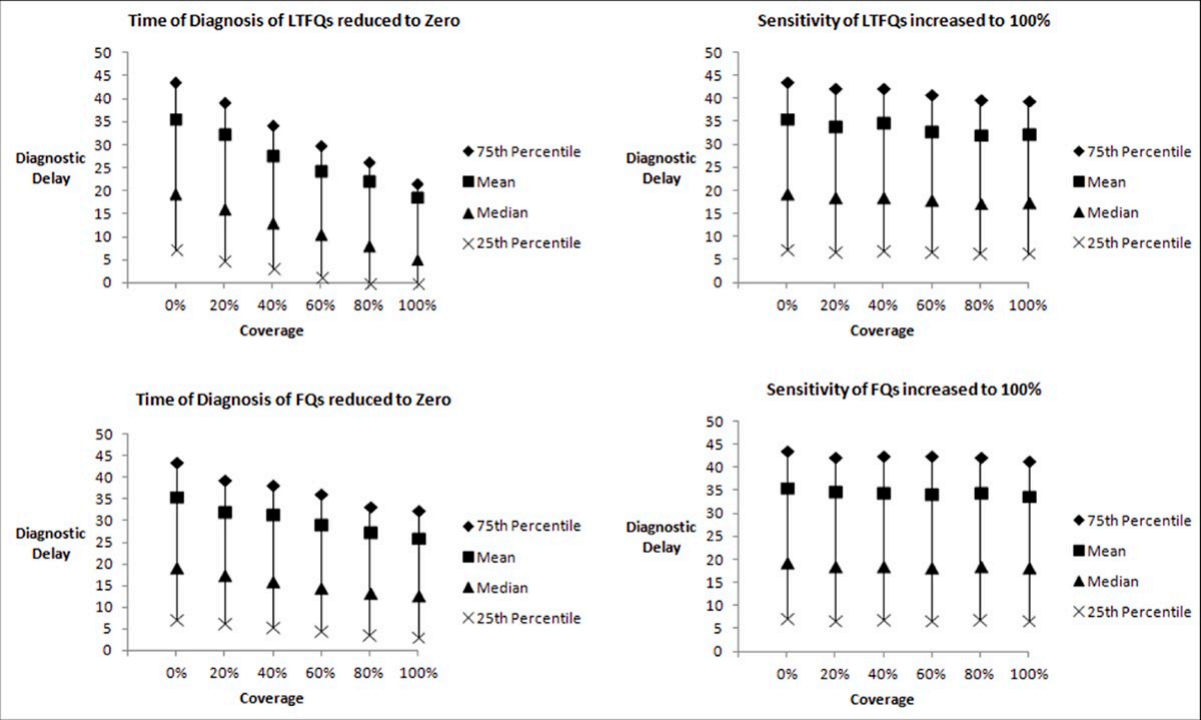
Effect of reduced time to diagnosis and increased diagnostic accuracy: Mumbai. Distribution of 10,000 simulations where time to diagnosis was minimized, and diagnostic accuracy (sensitivity) was maximized, for less-than-fully-qualified providers (LTFQs) and fully qualified providers (FQ). Coverage is the proportion of providers for whom the parameter specified was manipulated.

**Fig 3:**
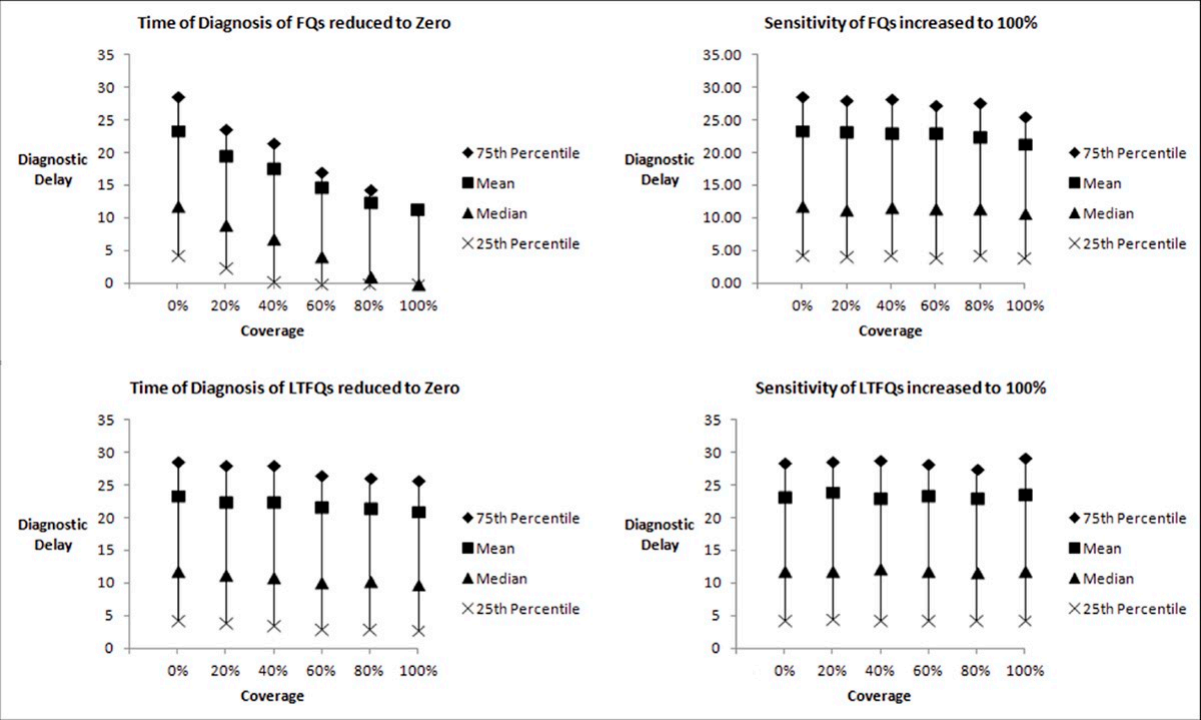
Effect of reduced time to diagnosis and increased diagnostic accuracy: Patna. Distribution of 10,000 simulations where time to diagnosis was minimized, and diagnostic accuracy (sensitivity) was maximized, for less-than-fully-qualified providers (LTFQs) and fully qualified providers (FQs). Coverage is the proportion of providers for whom the parameter specified was manipulated.

The results of counterfactual simulations for Mumbai and Patna are shown in Fig 2 and Fig 3, respectively. In Mumbai, reducing the time to diagnosis for LTFQs to 0 reduced the average diagnostic delay from 35.53 days (95% CI: 34.60, 36.46) to 18.72 days (95% CI: 18.01, 19.43) as provider coverage increased from 0% to 100%. Reducing the time to diagnosis for FQs to 0 reduced the average diagnostic delay from 35.53 (95% CI: 34.60, 36.46) to 26.01 days (95% CI: 25.23, 26.78) as provider coverage increased from 0% to 100% (Fig 2). In Patna, reducing the time to diagnosis for FQs to 0 reduced the average diagnostic delay from 23.39 days (95% CI: 22.77, 24.02) to 11.16 days (95% CI: 10.52, 11.81) as provider coverage increased from 0% to 100%. All these reductions in diagnostic delay were statistically significant (2-sample *t* test; *p* < 0.001) (Fig 3). Increasing the diagnostic accuracy of all LTFQs in Mumbai and all FQs in Patna did not lead to a statistically significant reduction in diagnostic delay (2-sample *t* test; *p* = 0.19 and *p* = 0.49, respectively). Reducing time to diagnosis for all private providers to that of public providers reduced diagnostic delay from 35.53 to 27.90 days in Mumbai and from 23.30 to 16.68 days in Patna (S1 Fig).

## Discussion

Several studies have documented the long and complex pathways of TB patients, characterized by provider shopping and diagnostic delays [10,18,20,21]. However, these studies are unable to provide estimates of the effects of underlying behavioral characteristics of providers (delaying the ordering of TB-specific diagnostic tests) and patients (switching across providers before receiving a diagnosis) that combine together to produce these delays. In this study, we overcame this challenge using a 2-pronged approach. First, we developed one of the first comprehensive discrete event simulation models for TB diagnostic pathways that explicitly includes these components, including providers’ time to diagnosis, patients’ time to switching, and probabilities of transitioning between different provider types. Second, we calibrated our model with detailed data codified from detailed interviews of population-based representative samples of TB patients in 2 Indian cities, Mumbai and Patna. We found that immediate testing for TB by LTFQs at the first visit (at the current level of diagnostic accuracy) could reduce the average diagnostic delay in Mumbai from 35.53 days (95% CI: 34.60, 36.46) to 18.72 days (95% CI: 18.01, 19.43). Similarly, immediate testing by FQs at the first visit (at the current level of diagnostic accuracy) could reduce the average diagnostic delay in Patna from 23.39 days (95% CI: 22.77, 24.02) to 11.16 days (95% CI: 10.52, 11.81). Improving the diagnostic accuracy of providers, per se, without reducing the time to testing, was not predicted to lead to any reduction in diagnostic delay.

Our results from Mumbai show that LTFQs take a longer time to order a TB test and experience a shorter time to patients switching away from them compared to FQs and public providers. Recent ethnographic studies of LTFQs may provide a qualitative understanding of these quantitative findings. The longer time to diagnosis may be attributed to prolonged use of medications to provide symptomatic relief to patients, a desire to manage illness cost effectively by avoiding prescription of expensive tests, uncertainty about the presentation of TB, and lack of awareness or uncertainty about the accuracy of available TB tests, among other reasons [17]. The shorter time to patient switching is suggestive of lower willingness to remain in care at LTFQs compared to at FQs (although we cannot directly estimate this willingness with our data and modeling approach), reflecting patients’ perception of LTFQs’ limited ability and the lower criticality of symptoms in earlier stages of care seeking [9]. These findings together also provide a quantitative basis for the widely acknowledged fact that first consultation with informal providers (LTFQs) is one of the strongest risk factors for delayed TB diagnosis [10,18].

Interestingly, after accounting for providers’ time to diagnosis and patients’ time to switching, which together determine the probability of a patient receiving a diagnosis, we find that the diagnostic accuracy of FQs and public providers is very high and not very different from each other in both Mumbai and Patna. These providers are typically visited later in the care seeking pathway, by which point patient symptoms may have become more apparent, thereby making diagnosis easier. Moreover, patients in India typically carry their medical records from previous consultations, and inspecting them may help these providers rule out other conditions and focus on TB. This finding suggests that uptake of novel, more accurate diagnostic technologies such as GeneXpert, which are typically employed by FQs and in the public sector, might have limited impact on diagnostic delay and TB incidence.

Combining operational models of health systems with transmission models of disease dynamics is important to obtain more accurate and realistic estimates of the potential impact of new diagnostic technologies, although very few studies have done this successfully [24]. One study used a combined model to demonstrate the interaction between operational elements of the healthcare system: The impact of a new, more accurate diagnostic tool is higher if accompanied by reduced patient delay and increased access to treatment [25]. Another study showed that substituting smear microscopy with a hypothetical test of higher sensitivity and lower sample burden could reduce TB incidence through reduced laboratory turnaround times [26]. However, these studies use simplified operational models that focus only on a limited set of activities in the care seeking pathway (e.g., waiting of the patient at the diagnostic center, sputum collection, and multiple visits to the diagnostic center for diagnosis and treatment) and focus exclusively on the public health system [25–27]. As a result, they cannot be used to evaluate whether more accurate diagnostic tests would reduce the extent of provider shopping (due to failed diagnosis) in the private sector, and thereby reduce the overall diagnosis delay.

Transmission models have incorporated provider shopping behavior by developing patient care pathways that span public and private sectors [28,29]. One study considers 2 types of providers (public and private) and assumes that patients seek care with each type at a constant rate, which is approximated using data from earlier pathway studies. However, this study does not explicitly consider switching between these 2 types of providers [29]. Another model incorporates such switching behavior but calibrates the model using data from a non-representative sample of patients enrolled for treatment in the public sector (DOTS centers in Delhi) only [9]. Comparing our results to these previous studies shows that pathways followed by patients who reach the public sector for treatment are markedly different from those of patients who remain in the private sector. For instance, in the Delhi study [9], the probability of patients accessing DOTS centers in Delhi as their first point of contact was 0%, and the probability of transitioning from an informal private provider to a public provider was less than 5%. In contrast, our estimates of these parameters obtained using data from a more representative household survey of TB patients treated in both the private and public sector are higher. Even in Patna, with a weak public system, the chance that a TB patient first approached the public sector was 6%, and the chance that they switched from an LTFQ to the public sector was 50%. As a result, the cost-effectiveness of increasing referrals from LTFQs to the public sector in reducing TB incidence might be lower than estimated in [28]. Similarly, another study developed a simplified model of patient pathways consisting of a constant probability of remaining undiagnosed at the end of each stage of consultation and a set of probabilities of switching from the private sector to the public sector that depended on the stage of consultation [22]. It assumed the former to be 70% and estimated the latter using data on patients enrolled for treatment in the public sector in Bangalore. The study found that reducing the failure rate by 50% at each consultation would reduce TB transmission by 43%. Estimation results of our more comprehensive model show that public providers and FQs have a failure rate of less than 0.4 (the probability of a correct diagnosis is more than 0.6) and that there is a non-trivial probability of patients switching from the public to the private sector. Consequently, the impact of measures that aim to reduce the failure rate in the private sector might be lower based on our model estimates compared to those in [22]. Overall, our results highlight that care pathways are likely to be very different based on the specific context (Mumbai and Patna in our case), and hence extreme caution must be exercised in using simplified pathway models to estimate the impact of new diagnostic technologies at a national level. In fact, our results suggest that a locally optimized intervention strategy, targeting the relevant mix of providers for each region (e.g., district, state) may be more effective than a single intervention strategy for the entire country.

All the above studies include a single parameter—probability of successful diagnosis (or, equivalently, failure rate)—that combines diagnostic accuracy at each stage with the probability of receiving a diagnosis at that stage. This is problematic because the former depends on the specific method and technology used for diagnosis whereas the latter depends on provider behavior (time to diagnosis) and patient behavior (time to switching). Because of the rich data collected from the patient interviews, our model is actually able to separately estimate these 2 components. This distinction enables us to compare the impact of reducing the time to diagnosis of a provider—thereby increasing the probability of diagnosis (S1 Text contains the mathematical expression for the relationship between time to diagnosis and probability of diagnosis)—with the impact of increasing the diagnostic accuracy of the provider. We find that improving the diagnostic accuracy of providers, without reducing their time to diagnosis, does not lead to a significant reduction in diagnostic delay. In contrast, reducing the time to testing by providers, without improving their diagnostic accuracy, leads to a significant reduction in diagnostic delay.

These findings can inform the operational design of private provider engagement models for TB control. Recent efforts in 2 Indian cities, Mumbai and Patna, have focused on incentivizing and subsidizing FQs to order more accurate diagnostic tests (GeneXpert or CBNAAT), notify cases, and provide free anti-TB drugs [30]. If the goal is to reduce diagnostic delay and incidence in the long run, our results suggest that these efforts should be complemented with promotion of early use of screening tests (combined with referrals by LTFQs in Mumbai). Furthermore, with regards to development of new diagnostic tests, our findings provide additional operational support for inexpensive point-of-care screening tests, which can generate a referral from LTFQs to FQs to counter the switch of patients from one LTFQ to another [31,32].

Our study has some limitations emanating from the method and context of data collection. First, the pathway surveys were conducted in 2 Indian cities, Mumbai and Patna. Unfortunately, we did not have access to data on key parameters such as prevalence of TB and number of providers for these 2 cities, thereby making it difficult to extrapolate our findings to other urban centers. However, the contrast between these 2 settings (Mumbai has a much stronger public health system but also a strong presence of LTFQs, whereas Patna has a weak public health system and a preponderance of FQs [21,33,34]) suggests that our estimates could represent a plausible range of parameter values that are likely to hold for other cities. Second, it is well known that patient pathway surveys are subject to recall bias [35]. The study was designed to mitigate this recall bias by triangulating information available in the patient records (e.g., dates), wherever possible. Interviews were conducted in the presence of family members (parents/spouse/children), and calendars with relevant dates including holidays and religious festivals were used to aid recall. However, it is plausible that the actual diagnostic delays, and consequently the magnitude of the actual reduction in delays, may be substantially greater than those predicted from our analysis. In that regard, our model results should be taken as conservative estimates. Third, patients were included in the pathway survey and interviews if they self-reported being on anti-TB treatment, but they were not tested using a gold standard TB test such as liquid culture. Given the toxicity of TB treatment and concomitant side effects, and its lack of efficacy against other conditions, it is unlikely that patients would continue anti-TB treatment if they did not have TB. Fourth, we assumed that model parameters do not change across consultation stages, which might not be completely realistic. For instance, rate of diagnosis and diagnostic accuracy may be higher in later stages as providers benefit from information recorded in the earlier stages and from more pronounced symptoms. We estimated a variant of the model with stage-dependent parameters for Mumbai (S2 Table), but we could not obtain consistent estimates for Patna due to the smaller sample size (64 patients). Fifth, we assumed that the switching behavior of patients is independent of provider behavior to simplify the estimation procedure. Future qualitative studies are needed to inform the validity of this assumption. In accordance with previous studies of patient pathways, our sample size was limited due to the intensive nature of the surveys and patient interviews. However, compared to previous studies [10], the data used to calibrate our modeling approach [20,21] have a few important advantages. First, despite the small sample size, the data are representative of the population in the respective cities due to a rigorous enumeration and sampling approach, which is described in [20,21]. Second, the sample consists of patients who were treated in the public as well as private sector, in contrast to many previous studies that started with patients in the public sector and traced their pathways back [9].

Our study can be extended in 2 directions. First, as indicated earlier, LTFQs need to be encouraged to order screening tests earlier and to refer patients to FQs. However, in the absence of explicit incentives, this behavior is likely to lead to a potential loss of income for LTFQs. Further studies are required to quantify the incentives for early screening and referral based on the public health benefit that might accrue from overall reduction in diagnostic delay. Second, our model needs to be embedded in transmission models to accurately translate the reduction in diagnostic delay into a reduction in TB disease burden in terms of prevalence and incidence [36].

## Supporting information

S1 Checklist STRESS-DES checklist for reporting discrete event simulation studies. (XLSX)

S1 Codebook Quantitative codebook for in-depth patient interviews. (DOCX)

S1 Fig Equalizing time to diagnosis of private and public providers. (TIFF)

S1 Table Rates of diagnosis and switching. (DOCX)

S2 Table Stage-wise estimates of rate of diagnosis and rate of switching: Mumbai. (DOCX)

S3 Table New patients: Probabilities of first consultation and switching across provider types. (DOCX)

S4 Table New patients: Diagnostic accuracy, probability of receiving a diagnosis, and probability of receiving a correct diagnosis. (DOCX)

S5 Table New patients: Rate of diagnosis and switching. (DOCX)

S1 Text Model formulation. (DOCX)

S2 Text Model validation against held-out sample. (DOCX)

## Abbreviations

FQ: fully qualified provider
LTFQ: less-than-fully-qualified provider
TB: tuberculosis
