# Supplementary material for: Predicting the impact of patient and private provider behavior on diagnostic delay for pulmonary tuberculosis patients in India: A simulation modeling study
Source: PLoS Med. 2020 May 14;17(5):e1003039. doi: 10.1371/journal.pmed.1003039 (PMC7224455; doi:10.1371/journal.pmed.1003039)
Supplement: S1 Codebook — (DOCX) [file pmed.1003039.s002.docx]

**Patient Pathway to Tuberculosis Care in Mumbai/Patna.**

**In-depth Interview-Quantitative data code sheet**

**THE FOUNDATION FOR MEDICAL RESEARCH**

In collaboration with Sambodhi Research & Communications Pvt. Ltd. and Bill and Melinda Gates Foundation.

**18^th^May2014**

| **Identification no: 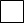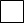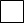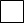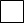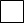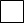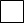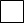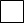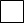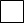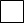**  **Respondent code: 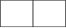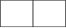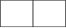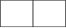** | | | |
| --- | --- | --- | --- |
| State name: _________________________________ State no : 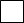 | | | |
| Ward name:­­­­­­­­­­­­­­­­­­­­_________________________________ Ward no : **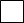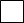** | | | |
| Cluster no: **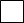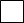** Household no: **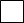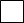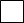** | | | |
| Name of the 1^st^ interviewer: ____________________________  Name of the 2^nd^ interviewer: ___________________________ | | | |
| Type of case: A6_3 _______________ ______ | | | |
| **No. of visits** | **Interview status** | | **Interview Time** |
| - First visit   DD MM YY  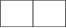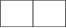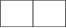 | - Incomplete | - Complete | S.T ______:______  E.T______:_____ |
| - Second visit   DD MM YY  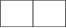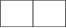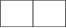 | - Incomplete | - Complete | S.T______:______  E.T______:___ |
| - Third visit   DD MM YY  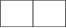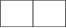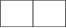 | - Incomplete | - Complete | S.T ______:______  E.T______:______ |
| Location of the interview: | | | |

| **Section1**  **Patient profile** | | | | | | |
| --- | --- | --- | --- | --- | --- | --- |
| **Q.no** | **Question** | **Response with Codes** | | | | **Code box** |
| B1_A | Age of the patient |  | | | | |
| B1_A1 | Age of the respondents  (If patient is not the respondent – in case of minor/aged patients) |  | | | | |
| B1_B | Gender of the patient | Male=1 | Female=2 | | |  |
|  |  | Transgender=3 | | | |  |
| B1_B1 | Gender of the respondent  (If patient is not the respondent – minor/aged) | Male=1 | | | Female=2 |  |
|  |  | Transgender=3 | | | |  |
| B2 | Education | Illiterate=0 | Literate but no formal education=1 | | |  |
|  |  | Primary (4^th^ or less than 4th standard)=2 | Secondary (9^th^ or less than 9th standard)=3 | | |  |
|  |  | Senior secondary (SSC/HSC)=4 | Graduate and above=5 | | |  |
| B3_1 | Nature of work (what kind of wok he/she does) | Unemployed=0 | Salaried=1 | | |  |
|  |  | Self-employed=2 | Daily wage worker/ Casual labourer=3 | | |  |
|  |  | House wife=4 | | | |  |
| B3_2 | No. of days in a month that he/she works |  | | | | |
| B4_1 | Marital status | Single=1 | Married=2 | | |  |
|  |  | Separated=3 | Divorced=4 | | |  |
|  |  | Widowed=5 | | | |  |
| B4_2A | No. ofmales in the household (age 15 and above) |  | | | | |
| B4_2B | No. of females in the household(age 15 and above) |  | | | | |
| B4_2C | No. children under 15 years in the household |  | | | | |
| B4_2D | No. children under 6 years in the household |  | | | | |
| B4_2E | No. of earning members in the household |  | | | | |
| B5 | Religion | Hindu=1 | Muslim=2 | | |  |
|  |  | Christian=3 | Buddhist=4 | | |  |
|  |  |  | | | |  |
| B6_1 | Mother tongue |  | | | | |
| B6_2 | Which language are you comfortable with? |  | | | | |
| B6_2A | Can he/she speak Hindi | Yes=1 | No=0 | | |  |
| B6_2B | Can he/she read Hindi | Yes=1 | No=0 | | |  |
| B6_2C | Can he/she write in Hindi | Yes=1 | No=0 | | |  |
| B6_3A | Can he/she speak Marathi | Yes=1 | No=0 | | |  |
| B6_3B | Can he/she read Marathi | Yes=1 | No=0 | | |  |
| B6_3C | Can he/she write in Marathi | Yes=1 | No=0 | | |  |
| B7_1 | Native place |  |  | | |  |
| B7_2A | Current address |  |  | | |  |
| B7_2B | How long resident in Mumbai/Patna |  |  | | |  |
| B8_1 | Does he/she (patient) consume any addictive substance(If No, skip to B9_1) | Yes=1 | No=0 | | |  |
| B8_2 | If yes, which? | Cigarettes/ beedi=1 | Alcohol=2 | | |  |
|  |  | Khaini/Tobacco chewing=3 | Injection drugs=4 | | |  |
|  |  |  | | | |  |
| B9_1 | (Patient) Suffering from any chronic condition(If No, skip to B10_1) | Yes=1 | No=0 | | |  |
| B9_2 | If yes, which? | HIV=1 | Diabetes=2 | | |  |
|  |  | Hypertension=3 |  | | |  |
| B10_1 | Electricity supply in the household | Own connection=1 | Shared connection=2 | | |  |
|  |  | Rent connection= 3 | None=0 | | |  |
| B10_2 | Type of toilet facility | no facility/bush/field=1 | shared pit toilet/Latrine=2 | | |  |
|  |  | own pit toilet/latrine=3 | | | |  |
| B10_3 | Source of drinking water in the household | Municipal=1 | bore hole=2 | | |  |
|  |  | Tanker=3 | public/Private well=4 | | |  |
|  |  |  | | | |  |
| B10_4 | Cooking fuel used in the household | LPG cylinder=1 | BIOGAS=2 | | |  |
|  |  | Chula=3 | Kerosene Stove=4 | | |  |
|  |  | Dung Cakes=4 |  | | |  |
| B10_5 | Number of household members per sleeping room |  | | | | |
| B11_1 | Type of windows | No=1 | Only frame=2 | | |  |
|  |  | With door=3 | Sliding doors=4 | | |  |
|  |  | Grilled=5 |  | | |  |
| B11_2 | Type of flooring | Tiled=1 | Koba/Dhalaiyya/Nahla=2 | | |  |
|  |  |  | | | |  |
| B11_3 | Material of exterior walls | Aluminium=1 | Concrete=2 | | |  |
|  |  | Mud=3 | Wood=4 | | |  |
|  |  | Tarpaulin=5 | |  | |  |
| B11_4 | Type of roofing | Concrete=1 | Asbestos=2 | | |  |
|  |  | Tin sheet=3 |  | | |  |
| B12_1 | Household ownership | Yes=1 | No=0 | | |  |
| B12_2 | Ownership of a bank or post-office account | Yes=1 | No=0 | | |  |
| B12_3 | Ownership of a mattress | Yes=1 | No=0 | | |  |
| B12_4 | Pressure cooker | Yes=1 | No=0 | | |  |
| B12_5 | Chair | Yes=1 | No=0 | | |  |
| B12_6 | Cot/bed | Yes=1 | No=0 | | |  |
| B12_7 | Table | Yes=1 | No=0 | | |  |
| B12_8 | Electric fan | Yes=1 | No=0 | | |  |
| B12_9 | Radio/transistor | Yes=1 | No=0 | | |  |
| B12_10 | Black and white television | Yes=1 | No=0 | | |  |
| B12_11 | Colour television | Yes=1 | No=0 | | |  |
| B12_12 | Sewing machine | Yes=1 | No=0 | | |  |
| B12_13 | Washing machine | Yes=1 | No=0 | | |  |
| B12_14 | Mobile telephone | Yes=1 | No=0 | | |  |
| B12_15 | Any other telephone | Yes=1 | No=0 | | |  |
| B12_16 | Computer | Yes=1 | No=0 | | |  |
| B12_17 | Refrigerator | Yes=1 | No=0 | | |  |
| B12_18 | Watch or clock | Yes=1 | No=0 | | |  |
| B12_19 | Motorcycle or scooter | Yes=1 | No=0 | | |  |
| B12_20 | Car | Yes=1 | No=0 | | |  |
| B12_21A | Do the children go to school (If No, skip to section 2) | Yes=1 | No=0 | | |  |
| B-12_21B | Children go to private school | Yes=1 | No=0 | | |  |
| B12_21C | Children go to public school | Yes=1 | No=0 | | |  |
| B12_21D | Mode of transport to school­­­­­­­­­­­­­­­ |  | | | | |

| **Section 2**  **Questions on Patient Health Seeking behaviour for the current episode of illness (Tuberculosis)/ chest symptoms** | | | | | |
| --- | --- | --- | --- | --- | --- |
| **Q.no** | **Question** | **Response with Codes** | | | **Code box** |
| C1 | First point of care | Public sector=1 | | Private sector=2 |  |
| C1_A | If public, why | Free treatment=1 | | Close to home=2 |  |
|  |  | Recommended by Family/ Friends  =3 | | |  |
|  |  | Recommended by Family Doctor=4 | | |  |
|  |  |  | | |  |
| C1_B | If private, why | Better quality of treatment=1 | | Poor prior experience in the public sector=2 |  |
|  |  | Family doctor=3 | | |  |
|  |  | Recommended by Family/ Friends=3 | | |  |
|  |  | Recommended by Family Doctors=4 | | |  |
| C2 | He/she aware of any signs and symptoms of TB | Yes=1 | | No=0 |  |
| C3 | Can TB be cured | Yes=1 | | No=0 |  |
| C4 | Date of 1st onset of symptoms | ____ ___/___ ___/ ___ ___ | | | |
| C5_A | What symptoms did he/she have prior to being diagnosed with TB? | Cough persistent for more than 2-3 weeks=1 | | Coughing up blood=2 |  |
|  |  | Fever=3 | | Weight loss=4 |  |
|  |  | Night sweats=5 | | Constant tiredness=6 |  |
|  |  | Loss of appetite=7 | | Chest pain=8 |  |
|  |  | Breathlessness/Difficulty in breathing=9 | | |  |
| C5_B | What did he/she think these symptoms were due to? | Allergy=1 | | Common cough=2 |  |
|  |  | Smoking=3 | | Weather=4 |  |
|  |  | Asthma=5 | | Tuberculosis=6 |  |
|  |  |  | | |  |
| C6_A | Was he/she worried about his/her symptoms? | Yes=1 | | No=0 |  |
| C6_B | Did he/she share his/her fear/seek advice about the symptoms from anyone? (If No, skip to C7) | Yes=1 | | No=0 |  |
| C6_C | If yes, whom? | Faith healer=1 | | Doctor acquaintance/ friend=2 |  |
|  |  | Relative=3 | | Knowledgeable person in the community=4 |  |
|  |  | Colleague/Employer=5 | | Pharmacist=6 |  |
|  |  |  | | |  |
| C7 | After the symptoms and before approaching the above person did he/she think of seeking medical advice | Yes=1 | No=0 | |  |
| C8_A | Did he/she prolong going to the doctor for some reason(If No, skip to section3) | Yes=1 | No=0 | |  |
| C8_B | Reason for prolonging | Time consuming to wait=1 | Too expensive=2 | |  |
|  |  | Distance to facility=3 | Thought it wasn't serious enough=4 | |  |
|  |  |  | | |  |

| **Section 3**  **Questions on Pathway to care for the current episode of illness**  **(Individual provider details to be asked to the patient)** | | | | | | | |
| --- | --- | --- | --- | --- | --- | --- | --- |
| **Provider** | | | | | | | |
| **Q.no** | **Questions** | **Response with Codes** | | | | | **Code box** |
| D_A1 | Name |  | | | | | |
| D_A2 | Qualification | MBBS=1 | | BAMS=2 | | |  |
|  |  | BHMS=3 | | BUMS=4 | | |  |
|  |  | Traditional healers/ Quacks/ IFPs=5 | | | | |  |
|  |  | MD=6 | | | | |  |
| D_A3 | Location |  | | | | | |
| D_A4 | Type | Municipal/Government hospital=1 | | Municipal Health post=2 | | |  |
|  |  | Private clinic=3 | | Private hospital or Nursing home=4 | | |  |
|  |  |  | |  | | |  |
|  |  | Clinic run by NGOs=5 | | Local chemist or pharmacy=6 | | |  |
| D_A5 | Date of approaching this provider | ___ ___/ ___ ___/ ___ ___ | | | | | |
| D_A6 | Reason for approaching this provider | Close to home=1 | |  | | |  |
|  |  | Had taken treatment from the provider earlier for some other illness=2 | | | | |  |
|  |  | Recommended by Family/ Friends=3 | | | | |  |
|  |  | Recommended by Family Doctor=4 | | | | |  |
|  |  | Recommended by Specialists =5 | | | | |  |
|  |  |  | | | | |  |
| D_B1 | Did the provider advise any tests?(if No, skip to D_B12) | Yes=1 | | No=0 | | |  |
| D_B2 | Which tests were advised? | Chest X-ray=1 | | Sputum examination=2 | | |  |
|  |  | Clinical examination=3 | | Blood tests=4 | | |  |
|  |  | ESR-5 | | CBC-6 | | |  |
|  |  | Ultrasonography=7 | | TBGold-8 | | |  |
| D_B3 | How long after the first consultation was the patient advised tests (in days)? |  | | | | | |
| D_B4 | Which tests did the patient actually undergo? | Chest X-ray=1 | | | | Sputum examination=2 | |
|  |  | Clinical examination=3 | | | | Blood tests=4 | |
|  |  | ESR-5 | | | | CBC-6 | |
|  |  | Ultrasonography=7 | | | | TBGold-8 | |
|  |  | None of the above=9 | | | |  | |
|  |  |  | | | | | |
| D_B5 | How many days after the tests were advised did patient actually undergo the tests? |  | | | | | |
| D_B6 | If sputum examination was conducted, how many sputum smear examinations done? |  | | | | | |
| D_B7 | Which laboratory were the tests conducted? |  | | | | | |
| D_B8 | Reason for conducting the tests at the mentioned laboratory | Recommended by the provider=1 | | Affordable=2 | | |  |
|  |  |  | | | | |  |
| D_B9 | Did the patient collect test result?(If No, skip to D_B12) | Yes=1 | | | No=0 | |  |
| D_B10 | After how many days of conducting the tests, did the patient receive the test results? |  | | | | |  |
| D_B11 | After how many days of receiving the test results, did the patient approach the provider? |  | | | | |  |
| D_B12 | Did the patient leave the provider any time during this period?(if No, D_C1) | Yes=1 | | No=0 | | |  |
| D_B13 | Reason for leaving the provider | Too expensive=1 | | Distance to facility=2 | | |  |
|  |  | Lack of available facilities/Poor quality of service=3 | | Behaviour of provider and other staff=4 | | |  |
|  |  | Time consuming to wait=5 | | | | |  |
|  |  |  | | | | |  |
| D_B14 | Date of leaving the provider | ___ ___/ ___ ___/ ___ ___ | | | | | |
| D_B15 | Total no. of visits made to the provider during this period  **(skip to D_G7)** |  | | | | | |
| D_C1 | Any medication/treatment given by the provider prior to/ without ordering the tests?(If No, skip to D_C4) | Yes=1 | | No=0 | | |  |
| D_C2 | How long was this treatment/medication continued for (days) |  | | | | | |
| D_C3 | What medications were you prescribed? (To be confirmed with the help of photographed prescription/blister packs/chemist bills) |  | | | | | |
| D_C4 | Did the patient leave the provider any during this period?(If No, skip to D_E1) | Yes=1 | | No=0 | | |  |
| D_C5 | Reason for leaving the provider | Too expensive=1 | | Distance to facility=2 | | |  |
|  |  | Lack of available facilities/Poor quality of service=3 | | Behaviour of provider and other staff=4 | | |  |
|  |  |  | | | | |  |
| D_C6 | Date of leaving the provider | ___ ___/ ___ ___/ ___ ___ | | | | |  |
| D_C7 | Total no. of visits made to the provider during this period **(skip to D_G7)** |  | | | | |  |
| D_E1 | Did the provider make any diagnosis?(If No, skip to D_E6) | Yes=1 | No=0 | | | |  |
| D_E2 | If yes, what was the diagnosis? | Tuberculosis=1 | MDR-TB=2 | | | |  |
|  |  | Acute respiratory illness=3 | Pneumonia=4 | | | |  |
|  |  | Viral=5 |  | | | |  |
| D_E3 | Who did the provider tell about the illness | Patient=1 | Family member/Friend=2 | | | |  |
|  |  | Both=3 |  | | | |  |
| D_E4 | How many days after the initial examination was the diagnosis made? |  | | | | | |
| D_E5 | How long (days) did it take to start the treatment after the diagnosis? |  | | | | | |
| D_E6 | Did the patient leave the provider any during this period?(If No, skip to D_F1) | Yes=1 | | No=0 | | |  |
| D_E7 | Reason for leaving the provider | Too expensive=1 | | Distance to facility=2 | | |  |
|  |  | Lack of available facilities/Poor quality of service=3 | | Behaviour of provider and other staff=4 | | |  |
|  |  | Time consuming to wait=5 | | | | |  |
|  |  |  | | | | |  |
| D_E8 | Date of leaving the provider | ___ ___/ ___ ___/ ___ ___ | | | | | |
| D_E9 | Total no. of visits made to the provider during this period  **(skip to D_G7)** |  | | | | | |
| D_F1 | Was the treatment started after diagnosis? (If No, skip to D_F13) | Yes=1 | | No=0 | | |  |
| D_F2 | How long was/is the entire treatment course as advised by the provider?(days) |  | | | | | |
| D_F3 | Was MDR-TB treatment given (if applicable)? | Yes=1 | | No=0 | | |  |
| D_F4 | What drugs were given (No. of drugs and injections taken) | D1: ________________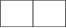 days  D2: ________________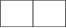 days  D3: ________________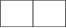 days  D4: ________________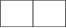 days  D5: ________________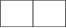 days  D6: ________________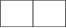 days  D7: ________________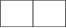 days  D8: ________________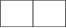 days  D9: ________________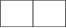 days  D10: ________________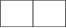 days | | | | |  |
| D_F5 | For how long were the drugs given/or have been taken so far? |  | | | | | |
| D_F6 | How often did/does the patient have to go to the provider to collect the drugs |  | | | | | |
| D_F7 | Was the patient ever hospitalized during the treatment?(If No, skip to D_F9) | Yes=1 | | No=0 | | |  |
| D_F8 | If yes, for how long?(days) |  | | | | | |
| D_F9 | Was there/is thereany improvement in patients’ health? | Yes=1 | | No=0 | | |  |
| D_F10 | Did he/she complete the entire treatment course(If Yes, skip to D_F13) | Yes=1 | | No=0 | | |  |
| D_F11 | If treatment not completed, why didn’t he/she complete the treatment? | Could not afford to purchase drugs anymore=1 | | Developed adverse drug reaction=2 | | |  |
|  |  | Started feeling better so stopped taking treatment=3 | | Treatment still ongoing=4 | | |  |
|  |  | Treatment ongoing=5 | | | | |  |
|  |  |  | | | | |  |
| D_F12 | What did he/she do after discontinuing the treatment? | Approached another provider immediately=1 | | Approached another provider after some period of time=2 | | |  |
|  |  | Started taking home remedies=3 | | Approached the same provider after some period of time=4 | | |  |
|  |  |  | | | | |  |
| D_F13 | Did the patient leave the provider any during this period?( If No, skip to D_G1) | Yes=1 | | No=0 | | |  |
| D_F14 | Reason for leaving the provider | Too expensive=1 | | Distance to facility=2 | | |  |
|  |  | Lack of available facilities/Poor quality of service=3 | | Behaviour of provider and other staff=4 | | |  |
|  |  | Time consuming to wait=5 | | | | |  |
|  |  |  | | | | |  |
| D_F15 | Date of leaving the provider | ___ ___/ ___ ___/ ___ ___ | | | | | |
| D_F16 | Total no. of visits made to the provider during this period  **(Move to next provider)** |  | | | | | |
| D_G1 | Did the provider give any advice/counselling related to TB(If No, skip to D_G3) | Yes=1 | | No=0 | | |  |
| D_G2 | What kind of information was given by the provider? | Dietary advice=1 | | Advice on adverse drug reaction=2 | | |  |
|  |  | Follow-up sputum test=3 | | Precautions for children under-6 years in the family=4 | | |  |
|  |  |  | | | | |  |
| D_G3 | Did the provider advice the patient to get tested for any other illness?(IF No, skip to D_G7) | Yes=1 | | No=0 | | |  |
| D_G4 | If yes, which? | HIV=1 | | Diabetes=2 | | |  |
|  |  |  | | | | |  |
| D_G5 | Did the provider give the respondent any specific advice regarding (if any) children under 6 in your household?(If No skip to D_G7) | Yes=1 | | No=0 | | |  |
| D_G6 | If yes, what advice? | Patient advised to get children tested for Tuberculosis=1 | | | | |  |
|  |  |  | | | | |  |
| D_G7 | Did the respondent go to another provider after this provider? | Yes | | No | | |  |

| - 1. **About how much did you spend during your interaction with each provider approached before you were diagnosed with TB till the time of receiving the TB treatment?/ About how much did you spend during your interaction with each provider that you approached since the onset of your chest symptoms?**   ( For all that don’t apply, mark N/A; Fill one line per visit) | | | | | | | | | | | | | | |
| --- | --- | --- | --- | --- | --- | --- | --- | --- | --- | --- | --- | --- | --- | --- |
| **Type of Provider**  (provider code)  E1_A | **No. of visits**  E1_B | **Total Time spent per visit**  (in hours, includes travel time)  E1_C | **Administrative Costs**  (consultative, registration)  E1_D | **Test costs**  (for sputum or other except x ray)  E1_E | **X ray or other imaging test (e.g. CT scan) costs**  **(**includes sending x rays to radiologist, travel & fees) E1_F | **Drug costs**  (all kinds total – TB plus other drugs such as cough syrups, vitamins)  E1_G | **Travel Costs**  [Patient(P)/Guardian (G)]  (return total) | | **Food costs**  [Patient(P)/Guardian (G)]  (total) | | **Accommodation Costs (if applicable)**  [Patient(P)/Guardian (G)]  (total) | | **Sub-Total costs per provider**  E1_K | **Insurance**  **Reimbursement**  (If yes: amount,  if no n/a)  E1_L |
|  |  |  |  |  |  |  | **P** E1_H1 | **G** E1_H2 | **P** E1_I1 | **G**  E1_I2 | **P** E1_J1 | **G**  E1_J2 |  |  |
| Provider 1 |  |  |  |  |  |  |  |  |  |  |  |  |  |  |
| Provider 2 |  |  |  |  |  |  |  |  |  |  |  |  |  |  |
| Provider 3 |  |  |  |  |  |  |  |  |  |  |  |  |  |  |
| Provider 4 |  |  |  |  |  |  |  |  |  |  |  |  |  |  |
| TOTAL |  |  |  |  |  |  |  |  |  |  |  |  |  |  |
| **Total Direct Pre-diagnostic& Diagnostic costs (sum sub-totals) minus insurance [**E1_M**] =** | | | | | | | | | | | | | | |

**Provider Code: 1-Municipal/Government hospital 2- Municipal health posts 3- Private MBBS Doctor Clinic 4-Private hospital or Nursing home**

**5- AYUSH practitioner 6- Traditional healer/Quacks/informal provide r 7- Clinic run by NGOs 8- Local chemist or pharmacy**

| **4.2** | **Treatment Costs (Report costs for the most recent anti-TB treatment)**  *****Not Applicable for chest symptomatic*** | | | | | | | | |
| --- | --- | --- | --- | --- | --- | --- | --- | --- | --- |
| **Q.no** | **Questions** | | **Response with Codes** | | | | | | **Code box** |
|  | **Costs related to DOT** | | | | | | | | |
| E2_A | Where does he/she/ did he/she currently take their TB drugs? (If home, go to E2_B1) | | Health facility/Hospital=1 | Home=2 | | | | |  |
|  |  |  | Community=3 | Workplace=4 | | | | |  |
|  |  |  | Dispensary=5 |  | | | | |  |
|  |  |  |  | | | | | |  |
| E2_A2 | How many times per week did he/she /does he/she go there place to take your drugs? | |  | | | | | | |
| E2_A3 | How long did/does it take you to get there  (one way) | | minutes walking _______________ | | | | | minutes with transport___________ | |
|  |  |  | Other | | | | | | |
| E2_A4 | How long did/does one of these visits take on average, including time on the road and waiting time (total turnaround time)? | | Hours | | | | | | |
| E2_A5 | From your home to the DOT place, how much did/ does it cost if you take transport? (both ways) | |  | | | | | | |
| E2_A6 | How much did / do you spend on food on the road, while waiting, for lunch? | |  | | | | | | |
| **Costs related to picking up the TB drugs – where drugs were/ are currently picked up** | | | | | | | | | |
| E2_B1 | How often did/ do you travel to the health facility / hospital for picking up your TB drugs? | | times / month | | | | | | |
| E2_B2 | How long did/does it take you to get there (one way) | | minutes walking_______ | | | | minutes with transport______ | | |
|  |  |  | Other ________________________________________ | | | | | | |
| E2_B3 | How long did/ does one of these visits take on average, including time on the road and waiting time (total turnaround time)? | | Hours | | | | | | |
| E2_B4 | From your home to the facility, how much did/ does it cost if you take transport? (both ways) | |  | | | | | | |
| E2_B5 | If you go to a facility to pick up your drugs, how much did/ do you spend on food on that day? (On the road, while waiting, lunch etc.) | |  | | | | | | |
| E2_B6 | Did/Do you have to pay administration fees when picking up your TB drugs?  If No, go to E2_B8. | | Yes=1 | No=0 | | | | |  |
| E2_B7 | If Yes, how much? | |  | | | | | | |
| E2_B8 | Did/Do you have any accommodation costs when picking up your TB drugs?  If No, go to E2_C1 | | Yes=1 | No=0 | | | | |  |
| E2_B9 | If Yes, how much per month? | |  | | | | | | |
| **Costs related to follow up tests** | | | | | | | | | |
| E2_C1 | Did you ever have to go to the health facility in addition to your regular visits for follow up tests since the beginning of treatment? (If No, go to E2_D1) | | Yes=1 | | No=0 | | | |  |
| E2_C2 | If yes, how many times? | | _________Times | | | | | | |
| E2_C3 | If yes, did you have to pay any additional costs any time during the entire period? | | Yes=1 | | | No=0 | | |  |
| E2_C4 | If so, what kind of costs and how much? | |  | | | | | | |
| E2_C4A | Fees=_________________ | E2_C4B | Sputum test __________________ | | | | | | |
| E2_C4C | X-ray ____________________ | E2_C4D | TB Drugs ____________ | | | | | | |
| E2_C4E | Other Drugs ______________ | E2_C4F | Other __________ | | | | | | |
| E2_C4G | Total________________ | | | | | | | | |
| E2_C5 | How long did/ does one of these follow-up visits take on average, including time on the road, waiting time and tests (total turnaround time)? | | _______Hours | | | | | | |
| **Costs related to Drug side effects and their management** | | | | | | | | | |
| E2_D1 | Did you ever have to go to the health facility in addition to your regular visits for any drug related side effects/reactions since the beginning of treatment? (If No, go to 4.3) | | Yes=1 | No=0 | | | | |  |
| E2_D2 | If yes, how many times | |  | | | | | | |
| E2_D3 | If yes, did you have to pay any additional costs any time during the entire period? | | Yes=1 | No=0 | | | | |  |
| E2_D4 | If so, what kind of costs and how much? | |  | | | | | | |
| E2_D4A | Fees______________ | E2_D4B | Sputum test __________ | | | | | | |
| E2_D4C | X-ray_____________ | E2_D4D | TB Drugs_____________ | | | | | | |
| E2_D4E | Other Drugs_______________  ­­­­­­­ | E2_D4F | Other________________ | | | | | | |
| E2_D4G | Total______________________ | | | | | | | | |
| E2_D5 | How long did/ does one of these follow-up visits take on average, including time on the road, waiting time and tests (total turnaround time)? | | _______Hours | | | | | | |

| **4.3** | **Guardian/Accompanying person costs** | | | | | | |
| --- | --- | --- | --- | --- | --- | --- | --- |
| E3_A | Does/did any family/friend/DOT supporter accompany you on any visitsor go in your place to collect your TB drugs/medicines?(if No go to 4.4) | | Yes=1 | No=0 | |  | |
| E3_B | If Yes, on how many visits has your family/friend/DOT supporter accompanied you or gone in your place during your treatment period? | |  | | | | |
| E3_C | Costs during treatment per visit: | |  | | | | |
| E3_C1 | Transport ____________________ | E3_C2 | Food____________________ | | | | |
| E3_C3 | Accommodation__________________ | E3_C4 | Other(specify)__________________ | | | | |
| E3_C5 | Total ____________________ | | | | | | |
| E3_D | How much does the friend/family earn per day? | | 1.­­­­­­­­­­­­­­_________ | | 3.Doesn’t earn | |  |
|  |  |  | 2.Doesn’t earn | | 4.Don’t know | |  |
| E3_E | Why did someone accompany him/her? | | Distance=1 | | Security=2 | |  |
|  |  |  | Administrative barriers=3 | | Language barriers=4 | |  |
|  |  |  | Too ill to travel alone=5 | | | |  |
|  |  |  |  | | | |  |

| **4.4** | **Hospitalization** | | | | | | | |
| --- | --- | --- | --- | --- | --- | --- | --- | --- |
| E4_A | Have you ever been admitted to a hospital as an inpatient- for any of your TB symptoms prior to final TB diagnosis?/ For your chest symptoms?(if No go to 4.5) | | Yes=1 | No=0 | | |  | |
| E4_B | How many times have you been admitted to hospital for TB symptoms/chest symptoms? | |  | | | | | |
| E4_C | If Yes, how many days in total did you stay at the hospital? | |  | | | | | |
| E4_D1 | Hospital administration fees______________ | E4_D2 | Sheets/Linen:_____________ | | | | | |
| E4_D3 | Food (not provided by hospital)__________ | E4_D4 | Transport (return):________________ | | | | | |
| E4_D5 | Drugs:________________ | E4_D6 | Tests:______________ | | | | | |
| E4_D7 | Others:_______________ | E4_D8 | Total Costs:________________ | | | | | |
| E4_E | Did any family/friend stay with you while in hospital? | | Yes=1 | | No=0 | | |  |
| E4_F | If Yes, how many days did he/she stay with you (sleep there)? | |  | | | | | |
| E4_G | Were there any extra costs for the relative/friend for staying at the hospital? | | Yes=1 | | | No=0 | |  |
| E4_G1 | Accommodation (hospital or other)_________ | E4_G2 | Food_____________ | | | | | |
| E4_G3 | Transport________________ | E4_G4 | Other______________ | | | | | |
| E4_G5 | Total Costs_______________ | | | | | | | |
| E4_H | How much does the friend/family normally earn per day? | |  | | | | | |

| **4.5** | **Other Costs Food Supplements** | | | |
| --- | --- | --- | --- | --- |
| E5_A | Do you buy any food supplements for your diet because of the TB illness/ chest symptoms, for example  Vitamins, meat, energy drinks, juices, eggs or fruits? (if No, go to 4.6) | Yes=1 | No=0 |  |
| E5_B | What kind of items? | Fruits=1 | Drinks=2 |  |
|  |  | Vitamins/Herbs=3 | Meat=4 |  |
|  |  | Eggs=5 | |  |
|  |  |  | |  |
| E5_C | How much did you spend on these items in a month approximately? | ­­­­­­­­­­­­­­­­­­­­­­­­­­­­­ | | |
| E5_D | Has there been/was there any change in the share of total house expenditure on food due to your TB illness/chest symptoms?(if no, go to 4.6) | Yes=1 | No=0 |  |
| E5_E | What was the share of total house expenditure on food before your TB illness/ chest symptoms began? |  | | |
| E5_F | What was/is the share of total house expenditure on food during TB illness/ chest symptoms began? |  | | |

| **4.6** | **Other Illnesses** | | | | |
| --- | --- | --- | --- | --- | --- |
| E6_A | Do you have any chronic illness for which you are receiving treatment?(if No, go to 4.7) | Yes=1 | | No=0 |  |
| E6_B | If yes, which? |  | | | |
| E6_C | Are there any additional costs because of this other illness besides the costs that you have already mentioned? | Yes=1 | | No=0 |  |
| E6_D | If Yes, how much are these additional costs on an average per month? |  | | | |
| E6_D1 | Tests= =___________________ | E6_D4 | Food=___________________ | | |
| E6_D2 | Drugs=___________________ | E6_D5 | Other=___________________ | | |
| E6_D3 | Transport=___________________ | E6_D6 | Total Costs=___________________ | | |
| E6_E | How much did you spend on healthcare on average per month BEFORE the TB illness/ chest symptoms began? |  | | | |
| E6_F | How much did you spend on healthcare on average per month during the TB illness/ chest symptoms began? |  | | | |

| **4.7** | **Insurance** | | | |
| --- | --- | --- | --- | --- |
| E7_A | Do you have any kind of private or government health/medical insurance scheme? (If No. go to 4.8) | Yes=1 | No=0 |  |
| E7_B | If yes, what type? | Reimbursement scheme=1 | Monthly medical allowance=2 |  |
|  |  | Donor=3 | Family/community fund=4 |  |
|  |  | NGO=5 | |  |
|  |  |  | |  |
| E7_C | Have you received reimbursement for any costs related to the TB illness/chest symptoms?  **Cross-check with table.1 on pre diagnostic & diagnostic costs)** | Yes=1 | No=0 |  |
| E7_D | How much have you received as reimbursement? |  | | |

| **4.8** | **Coping Costs** | | | |
| --- | --- | --- | --- | --- |
| E8_A | Did he/she borrow any money to cover costs due to the TB illness/ current illness?(if No, go to E8_E) | Yes=1 | No=0 |  |
| E8_B | If yes, how much did he/she borrow? |  | | |
| E8_C | From whom did he/she borrow? | Family=1 | Neighbours/friends=2 |  |
|  |  | Relatives=3 | Private bank=4 |  |
|  |  | Cooperative=5 | |  |
|  |  |  | |  |
| E8_D | What is the interest rate on the loan? (%) | 1._____________ | | |
|  |  | I don’t pay any interest | | |
|  |  | I am not expected to pay back the money | | |
| E8_E | Has he/she sold any of their property to finance the cost of the TB illness/current illness?(if No, go to 4. 9) | Yes=1 | No=0 |  |
| E8_F | If yes, what did he/she sell? | Land=1 | Transport/vehicle=2 |  |
|  |  | Household item=3 | Farm produce=4 |  |
|  |  |  | |  |
| E8_G | What is the estimated market value of the property he sold? |  | | |
| E8_H | How much did he/she earn from the sale of their property? |  | | |
| E8_I | Did he/she use their personal savings to finance the cost of the TB illness/current illness? | Yes | No |  |
| E8_J | If yes, how much? |  | | |

| **4.9** | **Socioeconomic Information Individual Situation and Change in Income or Lost Productivity due to TB** | | | |
| --- | --- | --- | --- | --- |
| E9_A | Who is the primary income earner in the household? | Patient=1 | Wife/mother=2 |  |
|  |  | Husband/father=3 | Extended family=4 |  |
|  |  | Son/daughter=5 | |  |
|  |  |  | |  |
| E9_B | What is his/ her current employment status? | Salaried (go to E9_E1)=1 | Self-employed (go to E9_E1)=2 |  |
|  |  | Daily wage worker/casual laborer (go to E9_E1)=3 | On sick leave (go to E9_C1)=4 |  |
|  |  | Not employed (go to E9_C1)=5 | Retired (go to E9_C1)=6 |  |
|  |  | School, college (go to E9_J1)=7 | Homemaker (go to E9_J1)=8 |  |
|  |  |  | |  |
| E9_C1 | Is the reason for Not Working related to the TB illness/current illness? | Yes=1 | No=0 |  |
| E9_D | If yes, when was the last time he/she was working? (mm/yy) |  | | |
| E9_E1 | Has his/her work situation changed as a result of your TB symptoms/chest symptoms or seeking treatment for your TB symptoms/chest symptoms?(If No, skip to E9_J1) | Yes=1 | No=0 |  |
| E9_E2 | How did his/her work situation change? | No change in work=1 | Lost work/job(s) or had to quit work/job(s)=2 |  |
|  |  | Changed work(had to find a new job)=3 | Took new work in addition to current work/job(s)=4 |  |
|  |  |  | |  |
| E9_F | Has his/her monthly income changed because of his/her TB illness/current illness? | Yes- has increased=1 | Yes-has decreased.=2 |  |
|  |  | No change in income=3 | |  |
|  |  |  | |  |
| E9_G1 | What was his/her estimated personal take home earning per month BEFORE the TB illness/ current illness or before his/her chest symptoms began? |  | | |
| E9_G2 | What is his/her our estimated personal take home earning per month NOW? |  | | |
| E9_H1 | How many hours did he/she work on average per day BEFORE you became ill with TB/ or with the current chest symptoms? |  | | |
| E9_H2 | How many hours does he/she work on average NOW per day? |  | | |
| E9_I | Is the change related to the TB illness/ current illness? (if answer to E9_H2 is different from E9_H1) |  | | |
| E9_J1 | Has he/she ever stopped working/going to school/doing housework due to TB/ or his/her chest symptoms? (if No go to E9_K1) | Yes=1 | No=0 |  |
| E9_J2 | If yes, for how long? | Less than 1 month=1 | one month=2 |  |
|  |  | 2-3 months=3 | 4-5 months=4 |  |
|  |  | more than 6 months=5 | |  |
| E9_K1 | Did/Does someone stay home specifically to take care of him/her? (If No, go to E9_M ) | Yes=1 | No=0 |  |
| E9_K2 | If yes, for how long? |  | | |
| E9_L | Did they quit their income-earning job to stay home and care for the patient? | Yes=1 | No=0 |  |
| E9_M | Is someone doing the work that you used to do? | Daughter=1 | Son=2 |  |
|  |  | Spouse=3 | Friend=4 |  |
|  |  | Nobody=5 | |  |
|  |  |  | |  |
| E9_N1 | Does all of his/her children of school age attend school regularly?(If no, skip to E10_A­) | Yes=1 | No=0 |  |
| E9_N2 | If not, then why? | Needs to help around the house=1 | No money for school fees=2 |  |
|  |  | Also sick=3 | Has to work to earn income=4 |  |
|  |  |  | |  |

| **4.10** | **Household Income and Spending** | | | |
| --- | --- | --- | --- | --- |
| E10_A | How much did he/she estimate was the average income of his/her household per month BEFORE the TB illness/ current illness or before the chest symptoms began? | | | |
| E10_A1 | income patient |  | | |
| E10_A2 | income rest of household |  | | |
| E10_A3 | Other |  | | |
| E10_A4 | Total |  | | |
| E10_B | How much do you estimate is the average income of your household per month NOW? | | | |
| E10_B1 | income patient |  | | |
| E10_B2 | income rest of household |  | | |
| E10_B3 | Other |  | | |
| E10_B4 | Total |  | | |
| E10_C | Has the amount of food consumed per month changed due to the TB illness/ current illness or since chest symptoms began? | Yes- increased | 1 |  |
|  |  | Yes- decreased | 2 |  |
|  |  | No change | 0 |  |

| **Section 5**  **Checklist to be filled at the end of the interview**  **(items to be photographed at the end of the interview)** | |
| --- | --- |
| 1^st^ Provider | 2^nd^ Provider |
| - Any written notes by the provider: __ - Lab results or imaging results from the provider: __ - Prescriptions given by the provider: __ - TB medication blister packs: ___ - Other(specify):____________ | - Any written notes by the provider: __ - Lab results or imaging results from the provider: __ - Prescriptions given by the provider: __ - TB medication blister packs: ___ - Other(specify): ____________ |
| 3rd Provider | 4^th^ Provider |
| - Any written notes by the provider: __ - Lab results or imaging results from the provider: __ - Prescriptions given by the provider: __ - TB medication blister packs: ___ - Other(specify):_____________ | - Any written notes by the provider: __ - Lab results or imaging results from the provider: __ - Prescriptions given by the provider: __ - TB medication blister packs: ___    Other(specify):_____________ |
