# Supplementary material for: Predicting the impact of patient and private provider behavior on diagnostic delay for pulmonary tuberculosis patients in India: A simulation modeling study
Source: PLoS Med. 2020 May 14;17(5):e1003039. doi: 10.1371/journal.pmed.1003039 (PMC7224455; doi:10.1371/journal.pmed.1003039)
Supplement: S1 Table — (DOCX) [file pmed.1003039.s004.docx]

S2 Table: Rate of diagnosis and switching: Mean(sd)

|  | Rate of diagnosis $\left( \frac{\boldsymbol{1}}{\boldsymbol{\tau}_{\boldsymbol{d}}} \right)$ | | Rate of switching $\left( \frac{\boldsymbol{1}}{\boldsymbol{\tau}_{\boldsymbol{s}}} \right)$ | |
| --- | --- | --- | --- | --- |
|  | Mumbai | Patna | Mumbai | Patna |
| Public | 0.09 (0.01) | 0.18 (0.02) | 0.04 (0.00) | 0.01 (0.00) |
| FQ | 0.07 (0.01) | 0.10 (0.02) | 0.04 (0.01) | 0.06 (0.01) |
| LTFQ | 0.03 (0.01) | - | 0.05 (0.01) | 0.14 (0.06) |
| Chemist | - | - | 0.06 (0.01) | 0.12 (0.02) |

We assume that the time to getting a diagnosis from a provider (T_d_), and switching from a provider (T_s_), are exponentially distributed with rates $\left( \frac{\boldsymbol{1}}{\boldsymbol{\tau}_{\boldsymbol{d}}} \right),\left( \frac{\boldsymbol{1}}{\boldsymbol{\tau}_{\boldsymbol{s}}} \right)$ respectively.

Rate of diagnosis $\left( \frac{\boldsymbol{1}}{\boldsymbol{\tau}_{\boldsymbol{d}}} \right)$ is the average number of diagnoses per day per patient in consultation

Rate of switching $\left( \frac{\boldsymbol{1}}{\boldsymbol{\tau}_{\boldsymbol{s}}} \right)$ is the average number of switches per day per patient in consultation
