# Supplementary material for: Predicting the impact of patient and private provider behavior on diagnostic delay for pulmonary tuberculosis patients in India: A simulation modeling study
Source: PLoS Med. 2020 May 14;17(5):e1003039. doi: 10.1371/journal.pmed.1003039 (PMC7224455; doi:10.1371/journal.pmed.1003039)
Supplement: S2 Table — (DOCX) [file pmed.1003039.s005.docx]

S3 Table: Stage-wise estimate of rate of diagnosis and rate of switching (Mumbai)

|  | Rate of diagnosis $\left( \boldsymbol{1}/{\boldsymbol{\tau}_{\boldsymbol{d}}} \right)$  mean [95% CI] | | | | | Rate of diagnosis $\left( \boldsymbol{1}/{\boldsymbol{\tau}_{\boldsymbol{s}}} \right)$  mean [95% CI] | | | | | |
| --- | --- | --- | --- | --- | --- | --- | --- | --- | --- | --- | --- |
|  | **1** | **2** | **3** | **4** | **5** | **1** | **2** | **3** | **4** | **5** |  |
| Public | 0.03 [0.01,0.05] | 0.09 [0.03,0.15] | 0.1 [0,0.22] | 0.09 [0.09,0.09] | NA | 0.06 [0.03,0.08] | 0.05 [0,0.1] | 0 [0,0] | 0.18 [0.18,0.18] | NA |  |
| FQ | 0.02 [0,0.04] | 0.06 [0.03,0.09] | 0.21 [0.03,0.39] | 0.8 [0.8,0.8] | NA | 0.03 [0.01,0.05] | 0.05 [0.02,0.07] | 0.18 [0.01,0.34] | 0 [0,0] | NA |  |
| LTFQ | 0 [0,0] | NA | NA | NA | NA | 0.06 [0.02,0.1] | NA | NA | NA | NA |  |
| Chemist | 0.04 [0.02,0.07] | 0.17 [0.08,0.25] | 0.18 [0,0.38] | 0.6 [0.6,0.6] | 0.23 [0.23,0.23] | 0.03 [0.01,0.06] | 0.04 [0,0.08] | 0.24 [0,0.47] | 0 [0,0] | 0 [0,0] |  |
| Unknown | 0.03 [0.01,0.05] | 0.09 [0.03,0.15] | 0.1 [0,0.22] | 0.09 [0.09,0.09] | NA | 0.06 [0.03,0.08] | 0.05 [0,0.1] | 0 [0,0] | 0.18 [0.18,0.18] | NA |  |
