# Supplementary material for: Predicting the impact of patient and private provider behavior on diagnostic delay for pulmonary tuberculosis patients in India: A simulation modeling study
Source: PLoS Med. 2020 May 14;17(5):e1003039. doi: 10.1371/journal.pmed.1003039 (PMC7224455; doi:10.1371/journal.pmed.1003039)
Supplement: S3 Table — (DOCX) [file pmed.1003039.s006.docx]

S4 Table: New patients - Probabilities of first consultation and probability of switching across provider types

|  |  | Switching (π) | | | | | First consultation (α) | |
| --- | --- | --- | --- | --- | --- | --- | --- | --- |
|  |  | **Public**  **mean [95% CI]** | **FQ**  **mean [95% CI]** | **LTFQ**  **mean [95% CI]** | **Chemist** | **mean [95% CI]** | |  |
| **Public** | Mumbai  Patna | 0.57[0.29,0.86]  1.0[1.00 ,1.00] | 0.32[0.12,0.57]  - | 0.10[0.00,0.26]  - | -  - | 0.26[0.09,0.45]  0.06[0.00,0.24] | |  |
| **FQ** | Mumbai  Patna | 0.50[0.00,1.00]  0.24[0.08,0.42] | 0.25[0.00,0.68]  0.76[0.58,0.95] | 0.25[0.00,0.70]  - | -  - | 0.06[0.00,0.13]  0.61[0.49,0.73] | |  |
| **LTFQ** | Mumbai  Patna | 0.47[0.23,0.72]  0.50[0.00,1.00] | 0.53[0.28,0.79]  0.30[0.00,0.89] | 0.00[0.00,0.00]  0.20[0.00,0.68] | -  - | 0.55[0.40,0.70]  0.08[0.02,0.15] | |  |
| **Chemist** | Mumbai  Patna | 0.17[0.00,0.72]  0.44[0.22,0.71] | 0.50[0.16,0.90]  0.44[0.22,0.68] | 0.33[0.02,0.71]  0.11[0.00,0.00] | - | 0.14[0.05,0.24]  0.25[0.14,0.36] | |  |

Note: Sample size for this estimation, i.e., number of new patients in Mumbai is 43 and in Patna is 49.
