# Supplementary material for: Predicting the impact of patient and private provider behavior on diagnostic delay for pulmonary tuberculosis patients in India: A simulation modeling study
Source: PLoS Med. 2020 May 14;17(5):e1003039. doi: 10.1371/journal.pmed.1003039 (PMC7224455; doi:10.1371/journal.pmed.1003039)
Supplement: S4 Table — (DOCX) [file pmed.1003039.s007.docx]

S5 Table: New patients - Diagnostic accuracy, probability of receiving a diagnosis and probability of receiving a correct diagnosis

|  | Diagnostic accuracy (β)  mean [95% CI] | | Probability of receiving a diagnosis $\left( \frac{\boldsymbol{\tau}_{\boldsymbol{s}}}{\boldsymbol{\tau}_{\boldsymbol{s}}\boldsymbol{+}\boldsymbol{\tau}_{\boldsymbol{d}}} \right)$  mean [95% CI] | | Probability of receiving a correct diagnosis $\left( \boldsymbol{\beta*}\frac{\boldsymbol{\tau}_{\boldsymbol{s}}}{\boldsymbol{\tau}_{\boldsymbol{s}}\boldsymbol{+}\boldsymbol{\tau}_{\boldsymbol{d}}} \right)$  mean [95% CI] | |
| --- | --- | --- | --- | --- | --- | --- |
|  | **Mumbai** | **Patna** | **Mumbai** | **Patna** | **Mumbai** | **Patna** |
| **Public** | 0.90[0.83,0.97] | 1.00[1.00,1.00] | 0.62[0.52,0.72] | 0.96[0.94,0.97] | 0.56[0.47,0.65] | 0.96[0.94,0.97] |
| **FQ** | 0.90[0.81,0.98] | 0.95[0.89,1.00] | 0.71[0.60,0.83] | 0.64[0.53,0.75] | 0.64[0.54,0.74] | 0.61[0.51,0.72] |
| **LTFQ** | 0.72[0.45,0.99] | NA | 0.36[0.17,0.55] | 0.00[0.00,0.00] | 0.26[0.12,0.40] | NA |
| **Chemist** | NA | NA | NA | NA | NA | NA |

Note: Sample size for this estimation, i.e., number of new patients in Mumbai is 43 and in Patna is 49.
