# Supplementary material for: Predicting the impact of patient and private provider behavior on diagnostic delay for pulmonary tuberculosis patients in India: A simulation modeling study
Source: PLoS Med. 2020 May 14;17(5):e1003039. doi: 10.1371/journal.pmed.1003039 (PMC7224455; doi:10.1371/journal.pmed.1003039)
Supplement: S5 Table — (DOCX) [file pmed.1003039.s008.docx]

S6 Table: New patients - Rate of diagnosis and switching

|  | Rate of diagnosis  $\left( \frac{\boldsymbol{1}}{\boldsymbol{\tau}_{\boldsymbol{d}}} \right)$  mean [95% CI] | | Rate of switching  $\left( \frac{\boldsymbol{1}}{\boldsymbol{\tau}_{\boldsymbol{s}}} \right)$  mean [95% CI] | | Time of diagnosis $\left( \boldsymbol{\tau}_{\boldsymbol{d}} \right)$  mean [95% CI] | | Time of switching ($\boldsymbol{\tau}_{\boldsymbol{s}}\boldsymbol{)}$  mean [95% CI] | |
| --- | --- | --- | --- | --- | --- | --- | --- | --- |
|  | **Mumbai** | **Patna** | **Mumbai** | **Patna** | **Mumbai** | **Patna** | **Mumbai** | **Patna** |
| Public | 0.09[0.07,0.12] | 0.18[0.14,0.22] | 0.06[0.04,0.07] | 0.008[0.006 ,0.01] | 11.15[7.80,14.50] | 5.60[4.14,7.06] | 18.62[12.56,24.69] | 123.9[85.68,162.24] |
| FQ | 0.09[0.06,0.13] | 0.10[0.07,0.13] | 0.04[0.02,0.05] | 0.06[0.04,0.08]] | 11.00[6.42,15.59] | 9.99[6.67,18.16] | 23.63[14.63,32.63] | 18.22[10.57,25.87] |
| LTFQ | 0.03[0.01,0.05] | 0.00[0.00,0.00] | 0.05[0.03,0.07] | 0.14[0.04,0.27] | 41.68[0.0,175.06] | Inf | 20.87[08.26,33.49] | 11.58[0.00,180.01] |
| Chemist | - | 0.00[0.00,0.00] | 0.04[0.02,0.05] | 0.12[0.09,0.16] | - | Inf | 28.04[15.20,40.80] | 8.27[5.69,10.86] |

Note: Sample size for this estimation, i.e., number of new patients in Mumbai is 43 and in Patna is 49.
