# Supplementary material for: Predicting the impact of patient and private provider behavior on diagnostic delay for pulmonary tuberculosis patients in India: A simulation modeling study
Source: PLoS Med. 2020 May 14;17(5):e1003039. doi: 10.1371/journal.pmed.1003039 (PMC7224455; doi:10.1371/journal.pmed.1003039)
Supplement: S1 Text — (DOCX) [file pmed.1003039.s009.docx]

# S1 text: model formulation

## Model Parameters

j: Category of provider (1 – LTFQ, 2 – FQ, 3 – Chemist, 4 – Public)

1) π_j_ = Probability of choosing provider of j^th^ category

2) λ_d,j_ = Rate of Diagnosis of provider of j^th^ category

3) λ_s,j_ = Rate of Switching from provider of j^th^ category

4) Sens_j_ = Sensitivity of provider of j^th^ category

5) TPM_j, m_ = Probability of transitioning from provider of j^th^ category to provider of m^th^ category before getting diagnosed or after getting wrong diagnosis

## Probability of getting diagnosed when consulting provider of category j

Td_j_ and Ts_j_ denote the time of diagnosis and time of switching for provider of j^th^ category. We assume that Td_j_ and Ts_j_ follow exponential distributions with rates λd_j_ and μ_j_, respectively.

Time spent by any patient with the HCP of j^th^ category is min (Td_j_, Ts_j_).This time, therefore, follows an exponential distribution with rate λ_d,j_ λ_s,j_

Probability of diagnosis when consulting provider of category j = Probability that Td_j_<=Ts_j_

= $\int_{0} Prob\left( {Td}_{j}\leq{Ts}_{j} \right|{Td}_{j}=x)*Prob\left( {Td}_{j}=x \right)*dx$ = $\int_{0} e^{-{}_{s,j}*x}* {}_{d,j}e^{-{}_{d,j}*x}dx$

= $\frac{{}_{d,j}}{{}_{d,j}+{}_{s,j}}$

## Likelihood Function of the parameters, given the observations

N = Number of patients

Stages_i_ = Number of stages of consultation for the i^th^ patient

${Cat}_{i,j,k}=\left\{ \begin{aligned} 1,if the category of i^{th}patient at k^{th} stage of consultation is j \\ 0, otherwise \end{aligned} \right.$

$${Diag}_{i,k} =\left\{ \begin{aligned} 1, if {the i}^{th} patient gets some diagnosis(correct or incorrect) \\ at k^{th} stage of consultation \\ 0,otherwise \end{aligned} \right.$$

$${CDiag}_{i,k}=\left\{ \begin{aligned} 1,if the i^{th} patient is correctly diagnosed at k^{th} stage of consultation \\ 0,otherwise \end{aligned} \right.$$

$${Dur}_{i,k}=Number of days spent by i^{th} patient at k^{th} stage of consultation$$

Likelihood Function:

$$(\prod_{i=1}^{N} \prod_{j=1}^{4} {(_{j})}^{{Cat}_{i,j,1}})*(\prod_{i=1}^{N} \prod_{k=1}^{{Stages}_{i}} \prod_{j=1}^{4} {(_{d,j}e^{-\left( {}_{d,j}+{}_{s,j} \right){*Dur}_{i,k}})}^{{Cat}_{i,j,k}*{Diag}_{i,k}}{{(Sens}_{j})}^{{Cat}_{i,j,k}*{CDiag}_{i,k}}$$

$$\left( 1-{Sens}_{j} \right)^{{}^{{Cat}_{i,j,k}*{Diag}_{i,k}*\left( 1-{CDiag}_{i,k} \right)}}{(_{j}e^{-\left( {}_{d,j}+{}_{s,j} \right){*Dur}_{i,k}})}^{{Cat}_{i,j,k}*\left( 1-{Diag}_{i,k} \right)}$$

$$\prod_{m=1}^{4} {{(TPM}_{j,m})}^{{Cat}_{i,j,k}*{Cat}_{i,m,k+1}*(1-{CDiag}_{i,k})})$$
