# Supplementary material for: Predicting the impact of patient and private provider behavior on diagnostic delay for pulmonary tuberculosis patients in India: A simulation modeling study
Source: PLoS Med. 2020 May 14;17(5):e1003039. doi: 10.1371/journal.pmed.1003039 (PMC7224455; doi:10.1371/journal.pmed.1003039)
Supplement: S2 Text — (DOCX) [file pmed.1003039.s010.docx]

# S3 text: Model validation against held-out samplE

We adopted the following procedure for held-out validation:

1. Partition the dataset into training (90%) and testing (10%) datasets
2. Run the EM Algorithm on the training dataset and obtain the parameter estimates
3. Randomly allocate providers with unknown qualifications in the testing dataset as LTFQ/FQ using the estimates for proportions obtained from the training dataset.
4. In the training dataset, use raw data for the following components of the pathway at each stage of consultation: provider type visited, whether diagnosis was given and whether the diagnosis was correct.
5. Simulate time of diagnosis and time of switching at each stage of consultation using parameters estimated from training data and calculate the predicted time to diagnosis
6. Compare the predicted and actual time to diagnosis in the held-out sample
7. Repeat this experiment with different instances of training/testing datasets

Results from these experiments are shown in Table 1 below, which show that the 95% confidence intervals overlap in all experiments except one. These provide additional evidence regarding the validity of our modeling and estimation approach. We also conduct a pair-wise t-test and the p-values are reported in the table below.

Table 1: Results of out-of-sample model validation

| Held-out sample | 95% CI of actual time to diagnosis | 95% CI of predicted time to diagnosis | p-value |
| --- | --- | --- | --- |
| 1 | 20.88 [0,60.58] | 50.95 [0,109.50] | 0.334 |
| 2 | 42.75 [0,94.43] | 38.47 [19.07,57.54] | 0.858 |
| 3 | 55.75 [0,115.57] | 84.85 [18.22,151.48] | 0.455 |
| 4 | 31.75 [10.81,52.69] | 57.11 [29.16,85.06] | 0.110 |
| 5 | 22.88 [10.22,35.53] | 57.52 [37.19,77.85] | 0.005 |
| 6 | 13.75 [0,29.45] | 42.96 [9.66,76.27] | 0.009 |
| 7 | 49.88 [10.97,88.78] | 60.86 [12.58,109.14] | 0.682 |
| 8 | 26.00 [8.72,40.07] | 49.69 [22.68,76.70] | 0.110 |
| 9 | 57.20 [11.84,102.55] | 68.89 [17.75,120.04] | 0.753 |
